# Evaluation of an easy-to-use protocol for assessing behaviors of dogs retiring from commercial breeding kennels

**DOI:** 10.1371/journal.pone.0255883

**Published:** 2021-08-13

**Authors:** Shanis Barnard, Hannah Flint, Traci Shreyer, Candace Croney

**Affiliations:** Department of Comparative Pathobiology, Purdue University, West Lafayette, IN, United States of America; University of Lincoln, UNITED KINGDOM

## Abstract

Objective, reliable behavioral tests are needed to refine on-site welfare assessments of dogs housed at commercial breeding (CB) kennels and provide a basis to inform predictions of their behavior when retired from such kennels. This study tested the reliability, construct validity, and applicability of a protocol for the behavioral assessment of dogs from CB kennels that might be useful in comprehensive welfare assessments of this population. A sample of 447 dogs from 26 CB kennels in the Midwestern US were assessed in their pens. Responses to an approach test (performed on three consecutive days) and a behavioral reactivity test (e.g., traffic cone, toys, umbrella) were recorded. Results showed moderate to perfect (K_w_ = 0.51–1.00) inter-rater reliability between three independent observers. Approach test-retest analysis showed high correlation of approach test scores on days 1, 2 and 3 (r = 0.85, p<0.0001). Exploratory factor analysis extracted four main factors: Food Motivation (F1), Sociability (F2), Boldness (F3) (e.g., response to novel objects), and Responsiveness (F4) (e.g., response to an umbrella opening) confirming the ability of the test to measure behaviors of interest. All factors showed high internal consistency (Cronbach’s alpha 0.81–0.93) further supporting the robustness of the test construct. The demonstrated reliability of this protocol suggests that it may be usefully applied to assessing the behavior of dogs as a component of their welfare assessment in CB kennels. Doing so using even larger sample sizes may yield insights on the effects of housing and management practices on dog welfare while at the kennels, which may also help inform approaches that improve rehoming outcomes for retiring breeding dogs. Practical applications and limitations are outlined.

## Introduction

Large scale breeding of dogs is highly controversial, raising several welfare and ethical concerns that have been discussed in detail [see 1–3]. Chief among these include doubts about the capacity to meet the physical, behavioral, and mental needs of dogs in high volume kennels, and persistent concerns that profit is likely to be prioritized over dogs’ welfare needs in such establishments [1–3]. Problematically, the terms, ‘puppy mill’ and ‘commercial breeding’ continue to be conflated, confounding problem-solving given that the former term is subjective, has no legal definition, refers to establishments operating with no regulatory oversight, and can apply equally to small breeding establishments as a function of the care and conditions offered to dogs [1]. Nonetheless, the existence of commercial breeding kennels in the US reflects both the increasingly high demand for pet dogs that needs to be met, the inability of small-scale breeders, shelters and rescues to do so, and socio-ethical factors that support such enterprises, particularly when demand is high but supply is limited. These include the need to avoid infringements on the public’s right and expressed desire to retain choices in where they acquire dogs, inequities in accessing dogs that meet people’s preferred characteristics, and creation of black markets for dogs that further jeopardize their welfare [1, 4, 5].

A few papers based on owner reports have been published identifying risks associated with dogs thought to originate from commercial dog breeding kennels. These include higher rates of fear and health problems in former breeding dogs, as well as lower rates of trainability when compared with pet dogs reportedly not sourced from breeding facilities [3, 6, 7]. However, recent pioneering work carried out by this research group at commercial dog breeding facilities in the Midwest (US), found very low prevalence of health issues and only moderate prevalence of fear responses toward an approaching unfamiliar person among breeding dogs [8–10]. The differences in the two bodies of work warrant discussion. First, it is important to point out that, until recently, the only scientific literature available on the topic [2, 6, 7] relied on data sourced indirectly (i.e., the origins of the dogs assessed were not verified or traced back to the kennels they came from). These papers referenced unidentified commercial breeding establishments, often using terms such as ‘puppy farms’ or ‘mills’. The former term is typically used outside of the United States (US), and likely includes kennel types and management styles different from those used in United States Department of Agriculture (USDA)-licensed commercial kennels that were utilized in the on-site [6–8] studies. The problems with the latter term have already been noted. The owner-reported studies may, therefore, have included data from dogs kept in kennels where conditions could range from “modern, clean and well-kept to squalid, noxious and grave” [7]. Most importantly, direct in-kennel observations of the dogs involved were not conducted. In contrast, the on-site studies targeted the large network of legally operating and USDA inspected facilities, entirely excluding unlicensed breeders and those operating without any discernible regulatory oversight. Squalid conditions typically ascribed to ‘puppy mills’ are not permissible for USDA-licensed breeders in good standing, even for those operating at minimum standard for compliance. Also, it should be noted that because of the voluntary participation of breeders in the on-site studies, it is plausible that at least some of the kennels included operated at a higher level of care and welfare than the US average. However, currently, there is insufficient scientific information to gauge what average welfare standards might be across the entire US commercial dog breeding. Further, the recruitment criteria used in the on-site studies (such as USDA license, region of the country, dog weights and kennel sizes) would have applied to all legally operating breeders who met them. Thus, despite voluntary participation, it is unlikely that the kennels used were entirely anomalous within the broader target population or were any less representative than those included in the owner-report studies. In fact, because of the clearly defined study population and parameters for inclusion, as well as the verified status of the breeders, they are likely *more* representative of US commercial breeding populations than those relied on in earlier studies.

Despite the emerging divergence in study populations and findings related to the behavior and overall welfare of dogs in CB kennels, there is no debate that long-term confinement of dogs in sub-optimal conditions can severely impair their welfare [11–15]. Many assumptions about US CB facilities such as the lack of space, positive dog-human interactions, opportunities to exercise, and inadequate health care [6, 7] are not supported by empirical evidence and direct observations. Also unsupported are presumptions that all US CB kennels operate similarly and offer singularly low care and welfare standards, and that the conditions resemble those found in Europe and elsewhere. To facilitate accurate benchmarking of the current welfare conditions of dogs raised in legally operating CB facilities, reliable, science-based knowledge that can inform best practices for breeders and kennel owners is necessary.

Therefore, the aim of the present study was to develop and explore the reliability, construct validity, and potential applicability of a set of behavioral tests intended to be integrated into welfare assessments of dogs from commercial breeding kennels. The behavioral tests included an adapted version of the previously developed FIDO (Field Instantaneous Dog Observation) tool [10, 16, 17], and a newly developed behavioral reactivity test. For the purposes of this paper, the term ‘reactivity’ will be used to refer to the intensity, time, and type of reaction of dogs after exposure to a novel stimulus [18, 19].

The FIDO welfare assessment tool includes both a behavioral and a physical health assessment component. The physical health portion utilizes metrics such as body condition, cleanliness, coat condition, etc. [10, 16]. This portion of FIDO has been applied and validated elsewhere [10, 16], and therefore will not be the focus of this paper. The behavioral component, i.e., a human-approach test, was refined based on previous work [10, 17] and thus, needed new examination. The second portion of the assessment was a reactivity test loosely based on previously published work [20, 21]. The test was adapted and refined to suit dogs in commercial breeding kennels. These tests allowed, for the first time, the recording of levels of both social and non-social fear in this population of dogs by exposing them to unfamiliar people, a range of novel objects, and potentially startling sudden movements (e.g., opening an umbrella). Understanding the impact of social and non-social fear is key when assessing the behavior of dogs as behavior is one of three key components of animal welfare assessment [22].

Although there is a wide variety of behavioral tests in the literature (working dogs [23, 24]; shelter dogs [21, 25]; service dogs [26, 27]), these were developed with different dog populations and purposes in mind. The present study introduces for the first time a full behavioral test battery applied to a population of dogs from CB kennels. Such dogs are presumed to be raised in kennels with minimal or no exposure to novelty and/or unfamiliar people. For practical applicability in a commercial breeding setting, the test had to be short, easy to perform, and avoid any prolonged or unnecessary stress to the dogs, yet also capture individual behavioral variability.

For scientific integrity, when developing a new assessment tool, the researchers should provide evidence that the measures used are free from random errors, and that they are appropriate in measuring the attributes (i.e., in this case the behavioral responses) that they claim to be measuring [28]. Performing a comprehensive and robust validation process requires extensive resources and dedicated effort [28–30], and as reported by Patronek et al. [29], validity is not a fixed, single measurement event but rather a systematic process where the instrument properties are examined as a function of the specific context, population, and purpose in which they are administered. Hence, the current paper cannot claim validity, but instead uses a statistical approach to evaluate the reliability of the behavioral test used and its possible implications for behavioral assessments in this unique population of dogs.

To achieve this goal, and for comparison with previous work [20, 21], our specific objectives were to:

1. Assess inter-observer reliability (i.e., the level of agreement between different observers);
2. Assess test-retest reliability (i.e., the consistency in dogs’ responses to a repeated test across different days);
3. Assess construct validity (i.e., the goodness of the behavioral test, or if the test measures what it is designed to measure);
4. Assess internal consistency (i.e., the consistency within components designed to measure the same trait);
5. Evaluate the applied value of the behavioral assessment tool.

## Materials and methods

### Ethics statement

The procedures described were reviewed and approved by the Purdue University Institutional Animal Care and Use Committee (PACUC #1809001796). To protect the animals’ welfare and safety, if a dog showed extreme signs of fear or distress that could potentially harm the dog or the tester (such as persistent attempts to escape by scratching the floor or bumping the locked flap door that provided outdoor access, uninterrupted stereotypic activity or, overt aggressive responses to the tester such as teeth baring, growling or lunging) the test was stopped. Dogs were never handled, cornered, or forced to interact with objects or people. Permission to visit the kennels was granted by the owners and a consent form for participation in the research was signed prior to the commencement of the study.

### Subjects and facilities

The protocol was tested in 26 CB kennels located in the US Midwest (Indiana, Illinois, Ohio, and Iowa). Breeders with kennels were recruited for research participation through referrals from other participating breeders or through contacts within their communities, breeder education meetings and organizations, and other outreach activities. Participation in the study was voluntary. To be included in the study, kennels had to be legally operating and compliant with USDA regulations, and be large enough to provide at least 20 eligible study dogs (see inclusion criteria below). To ensure variability, no other restrictions on location, facility or management practices were applied during recruitment.

A total of 447 dogs were tested. All dogs were over 2 years of age (mean = 3.63, range 2–10 years old), and included 80.5% females (n = 360) and 19.5% males (n = 87) representing 47 different pure-breeds and designer crossbreeds (see Section 1 in S1 File for full list). Bitches in the last two weeks of gestation or those nursing puppies were excluded from the sample. Before our arrival, the breeder was asked to prepare a list of all the dogs in the kennel that met the inclusion criteria at the time of the visit. From that list, up to 20 dogs per facility were selected for testing using a random number generator (google.com). Depending on the facility, dogs were housed singly, in pairs, or groups (3–6 dogs per pen). Pens were different sizes (all exceeding USDA requirements, ranging from 0.7 to 4.5 m^2^, mean±SD 2.3±1.2 m^2^), as were flooring types/materials (e.g., concrete, tenderfoot). Some housing allowed for access to the outdoors (e.g., indoor only or with free indoor/outdoor access), and some pens had additional daily or weekly access to separate outdoor exercise yards.

### Procedures

All tests were performed in the indoor portion of each dog’s home pen, with the focal dog isolated and closed inside (if outdoor access was available). Before formal testing, a pilot study was conducted on a separate sample of 60 dogs to ensure that their responses to an approach test while closed inside and separated from their pen-mates did not differ significantly from those observed while inside as a group (see Section 2 in S1 File for details).

Dogs were left to acclimate to being closed in for three minutes before testing. Subjects received a behavioral assessment including the approach test (AT) with an unfamiliar person and the reactivity test (RT) described below. Because of the length of time required to complete testing of all dogs, testing at a single facility was spread across two consecutive days: all dogs received the AT on both days, while 50% of randomly selected dogs (using a random number generator in google.com) received the RT on day 1 of testing and the remaining dogs received it on the second day. All dogs received the RT immediately after the AT. A sub-sample of dogs (n = 201/447 from 16 facilities) was additionally visited a third time and received only the AT to assess the effect of test repetition over time (test-retest). To avoid any prolonged or unnecessary stress to the dogs, the RT was not repeated and therefore the assessment of a test-retest was possible only for the AT.

All tests were performed by the same two female experimenters trained on the use of the test and its standardization. To standardize the protocol administration, the training was performed at six pilot breeders not included in the study, whilst scorings were compared and discussed by reviewing the videos of dogs from pilot test sites until a unanimous understanding of definitions and an agreement was reached. The experimenter conducting the test verbally communicated the scores to a research assistant who stood out of sight and recorded all scores on a portable device (i.e., Microsoft Excel spreadsheet uploaded on an Apple iPad tablet). During the whole protocol, the researcher would maintain a sideways orientation relative to the dog, avoid direct eye contact, and perform the test with a soft posture and gaze. Unless the protocol specified differently, the researcher was instructed to remain silent, not to handle or pet the dog, and limit the interaction to handing the treats. Treats (1cm pieces of Pup-Peroni^®^, J.M. Smucker Co.) were sourced from a treat pouch fastened around the experimenter’s waist. The treats the breeders would normally use in the kennel were also kept on-hand in case the tested dog was disinterested in the ones we provided. All tests were scored live and recorded using a digital camera (Sony Handycam HDR-CX405, mounted on a tripod) for later reliability analyses. Time of testing varied between breeders as the research team had to accommodate the breeders’ schedule and availability, however, the time of day for testing at the same kennel was kept consistent across the two/three visits.

#### Approach Test (AT)

The dogs’ behavioral responses to an unfamiliar person approaching and initiating an interaction were assessed using a modified version of the Field Instantaneous Dog Observation Tool (FIDO; [10, 16]). Test-retest reliability assessment was performed by repeating the test over three consecutive days. The same experimenter tested the same dog on all three days. Because the subjects also received the RT either on day 1 or day 2, a possible effect of this confounding factor (i.e., the RT influencing the dogs’ response to the AT on the following day) was accounted for in the analysis.

The test followed a three-step protocol summarized below (see Section 3 in S1 File for detailed description). At each step, the dog’s response was recorded following the Red-Yellow-Green (RYG) scoring system (See Table 1). In brief, a dog was scored Red if it showed signs of fear, Green if it was undisturbed or affiliative in response to approach, and Yellow for ambivalent behavior or behavior that could not be characterized as clearly Red or Green.

**Table 1:** Description of Red-Yellow-Green (RYG) scoring system. RYG was used to assess the behavioral reaction of dogs during the three-step approach test.

Score	Description
Red = 0	• Fearful body language (e.g., ears back, whale eye, tail tuck, low and back posture)
	• Flight (e.g., increase distance, runs to the back of the pen, tries to escape)
	• Frozen or catatonic
	• Fight (e.g., barking, lunging, growling, hard and forward body language)
	• Stereotypic behaviors
Yellow = 1	• Ambivalent body language (e.g., lip-licking, head/eye aversion while approaching)
	• Ambivalent approach-avoid behavior
	• Ambivalent behaviors (e.g., behaviors are a mix of green and red)
	• Note: dog is not clearly red or green
Green = 2	• Relaxed body language (e.g., soft, loose, wiggly, neutral eyes/ears/posture)
	• Affiliative approach
	• Solicits attention (e.g., scrabbling at cage door or attempting to sniff/lick observer)
	• Neutral (undisturbed from behavior occurring prior to observer approach, e.g., eating/drinking, play, rest)

Step 1. Approach: An unfamiliar person (the experimenter) approached the pen and the immediate behavioral response of the dog was scored (RYG_app). The experimenter tossed a treat over the pen door, and whether the dog ate the treat or not was recorded (RYG_app/treat);

Step 2. Open door: The experimenter opened the door of the pen and the immediate behavioral response of the dog was scored (RYG_open). The experimenter offered a treat to the dog from their hand, and whether the dog took the treat from the hand or not was recorded (RYG_open/treat);

Step 3. Reach: The experimenter extended one hand toward the dog while visibly holding a treat in the other hand. The behavioral response of the dog to the experimenter reaching to touch him/her was scored (RYG_reach) and whether the dog allowed the experimenter to touch was recorded (TOUCH) along with whether or not the dog took the treat from the hand (RYG_reach/treat).

It should be noted that during the ‘touch’ action the researcher was instructed to extend their hand without stretching to reach or bending over the dog, and to touch the dog only if they approached the experimenter and allowed contact. If the dog showed signs of avoidance or fear/deflection (e.g., turning head away, avoiding gaze, freeze in place, whale eye) the dog was not touched, even if within reach.

#### Reactivity Test (RT)

The dogs’ responses when presented with small challenges were assessed by introducing a range of novel objects and brief social interactions during a short battery of tests. The battery included 10 subtests summarized in Table 2 (see also Section 4 in S1 File).

**Table 2:** Summary description of the subtests included in the reactivity test. A more detailed description of the test protocol and scoring system is presented in Section 4 in S1 File.

Subtest (time)	Summary description
1. Mat (30 sec)	Reaction to a rubber mat placed on the pen floor with a treat on top. The mat was left in the pen for the remainder of the test.
2. Leash (30 sec)	Reaction to a slip leash placed on one side of the mat with a treat on top. The leash was left there for the entire test to familiarize the dog with it before looping it over his/her head (see subtest 10).
3. Cone (30 sec)	Reaction to a plastic traffic cone placed on the mat. The cone and all subsequent objects were removed after scoring.
4. Problem solving (30 sec)	Success in retrieving (or not) a treat placed on the mat and under an upside-down bowl.
5. Squeaky toy	Initial and final reaction to the sound of a plastic squeaky dog toy squeezed in front of the dog for up to 10 times.
6. Ball toy (30 sec)	Reaction to a rubber ball placed on the mat.
7. Artificial dog (30 sec)	Reaction to a realistic dog statue placed on the mat.
8. Umbrella	Initial and final reaction to an umbrella opened in front of the dog for up to 10 times.
9. Commands	Reaction to the experimenter first calling the dog (‘*come command’*) and then luring him/her to sit using a treat (‘*sit command*’).
10. Loop leash	Reaction to the experimenter showing the leash in an open loop and attempting to slip the leash over the dog’s head. A treat was used to lure the dog inside the loop. The dog could retreat at any time as the loop was never closed around the dog’s neck.

All test props were prepared on a cart out of the dog’s sight. Each object was handed by an assistant to the researcher who would place it in the pen, then close the pen door and step back (at least 1 m away) maintaining a sideways orientation relative to the dog. The researcher would start the stopwatch and observe the dog’s behavior from afar. At the end of the exposure period, the object was removed, the door closed, and the next objects introduced. The squeaky toy and the umbrella were handled as described in Table 2 then taken away (not left in the pen). The total duration of the test was approximately 10 minutes.

The final selection of which items to introduce and which changes to apply compared to the original published work, was informed by a pilot study performed at 6 kennels (n = 60, not included in the study). The items were included to primarily trigger a social response towards people and conspecifics and an exploratory/avoidance response to novel/inanimate objects. We hypothesized that based on previous results with shelter and pet dogs [20, 21], responses to some items would reflect specific behavioral characteristics of interest. For example, the ball and squeaky toy previously proved to be a good measure of playfulness, the problem-solving task represented a measure of cognitive flexibility, the artificial dog was a proxy measure of intra-specific sociability, and the leash and commands served as a measure of trainability in dogs in shelters and homes [20, 21]. The cone was introduced as a purely novel object to assess the broader shyness/boldness component.

The dog’s response was scored live using a 3-point scale. While the explicit definitions for these scores varied by subtest (and can be found in Section 4 in S1 File under ‘scoring description’), in general, a score of 2 primarily reflected confident exploration/interaction with the stimuli or task; a score of 1 reflected cautious approach/interaction with the stimuli or task; and a score of 0 reflected fearful/aggressive responses to the stimuli, or failure to engage in the task.

### Statistical analysis

All statistical tests were carried out using IBM SPSS (v.24) software with α ≤0.05, unless otherwise specified. Based on previous work, a range of statistical tests commonly used to assess different test quality criteria were applied to reach our objectives [20, 21, 28].

#### Inter-observer reliability

The level of agreement between three independent observers was calculated by scoring 60 dogs (30% of the sample) from videos. This analysis was performed on each individual behavioral score from both the AT and the RT. All raters were female researchers who were previously trained on the protocols and had experience with its application in the field. Percentage of agreement and weighted Kappa values (K_w_) [31] were calculated for each observer pair combination and variable. For each variable, an average agreement across the three observers was also calculated through a mathematical mean. Agreement levels were interpreted on the basis of Landis and Koch ratings [32] as follows: K_w_ = 0.00, less than chance agreement; 0.01–0.20, slight agreement; 0.21–0.40, fair agreement; 0.41–0.60, moderate agreement; 0.61–0.80, substantial agreement; 0.81–0.99, almost perfect agreement; and 1, perfect agreement.

#### Test-retest reliability

An approach test score (AT_tot) was calculated by summing all individual scores from the three steps (max AT_tot score = 10). The consistency in the dogs’ responses to the approach test across the three days was calculated using Intraclass Correlation Coefficients (ICC, two-way mixed effect model, single measures reported); the 95% confidence interval for the ICC value is reported. To further explore if there was an effect of repetition over time, a Linear Mixed Model (LMM) was used. Normality and homoscedasticity of residuals were examined to ensure model assumptions were met (see Section 5 in S1 File for analysis of residuals). The possible effects of the RT being administered on the first or the second day of testing (RT_day) on the approach test scores was accounted for. The LMM used the AT_tot score as a dependent variable (treated as continuous), while the day, RT_day and their interaction as fixed effects, and dog subjects and facility as random effects.

Given that this was the first time this refined three-step version of the FIDO test was applied, we evaluated whether the behavioral response of the dog (measured using the RYG score, Table 1) changed as the interaction with the experimenter became progressively more intense (i.e., a person was standing behind the closed kennel door; the individual opened the door and stood passively; and finally, the person actively attempted to reach in and touch the dog). A Friedman test for repeated measures and post-hoc pair comparisons were used to assess whether the dogs’ RYG scores differed in response to each of the three steps of the test (i.e., approach, open door, and reach).

#### Construct validity

This step is used to determine the associations between test items developed to measure a specific trait, i.e., if different variables group together to measure one underlying construct of interest [28]. All variables from both behavioral tests were analyzed together to evaluate whether some items of the RT involving human interaction would group together into one dimension with the AT. Approach test scores consisted of day 1 of testing of all dogs. A common way of assessing construct validity in behavioral tests is to use a data reduction technique [28]. Since the test design used in the current study was considerably simplified compared to the tests used in the original papers [10, 20, 21] and the test dog population had not been previously assessed using this paradigm, it would have been unsound to make any a-priori assumptions about the underlying structure of our variables; therefore CFA (confirmatory factor analysis) was not deemed appropriate and an exploratory factor analysis (EFA) was chosen instead. The use of categorical variables with EFA is not ideal but possible [33]; thus, given the exploratory nature of this analysis, we ran the EFA in SPSS (which uses Pearson’s correlation matrices) by treating the variables as continuous. Limitations of taking this approach are discussed. EFA with varimax rotation and Eigenvalue >1 was used. Kaiser-Meyer-Olkin (KMO), communalities and Bartlett test of Sphericity were checked to ensure other data assumptions were met. EFA factor scores (standardized scores calculated using the least squares regression procedure in SPSS) were created to allow further analysis. Standardized scores represent the subjects’ placement on each factor and were preferred to a simple sum of the raw variables by factor as this former method takes into account the different weight (i.e., loading) that each item has on each factor [34].

*Effect of tester and RT_day*. A LMM was used to evaluate the possible effects of administration of the RT on either the first or the second day and the potential effect of different experimenters. The standardized factor scores calculated from the EFA were used as dependent variables with tester and RT_day included as fixed effects, and facility included as random effect. Analysis of residuals (provided in Section 6.1 in S1 File) showed that assumption of normality and homoscedasticity were acceptable.

#### Internal consistency

This criterion measured the consistency within components designed to measure the same trait. Cronbach’s alpha test was used to check the correlation within each dimension extracted by the EFA. Alpha coefficients greater than 0.70 were considered acceptable.

#### Applied value

To provide an example of how the results from the EFA can be used, the standardized factor scores generated for each dog were plotted using simple scatterplots. These plots show how the dogs tested in each facility scored with respect to the new behavioral components extracted by the EFA (e.g., sociability and boldness). These can be used to identify areas of concern within kennel populations that can be subsequently addressed. In the discussion, the value of these results and the potential of this behavioral assessment tool are explained.

## Results

### Inter-observer reliability

The percentage of agreement between observers ranged from 82–100% with K_w_ values ranging from moderate to perfect (Table 3). The lowest values were found for scores of the reaction to the artificial dog (82%, K_w_ = 0.51), which represented a moderate agreement.

**Table 3:** Agreement between observers.

	Ob 1-Ob 2		Ob 1-Ob 3		Ob 2- Ob 3		
Test	%	K_w_	%	K_w_	%	K_w_	Average Agreement
RYG_app	97	0.9	94	0.81	93	0.76	almost perfect
RYG_open	95	0.88	93	0.83	92	0.81	almost perfect
RYG_reach	96	0.91	92	0.81	92	0.8	almost perfect
TOUCH	100	1	96	0.91	96	0.91	almost perfect
Mat	93	0.9	97	0.91	93	0.81	almost perfect
Leash	90	0.73	93	0.84	87	0.68	substantial
Cone	93	0.89	92	0.79	87	0.72	substantial
Problem solving	95	0.88	96	0.9	95	0.85	almost perfect
Squeaky toy (initial)	97	0.91	96	0.91	93	0.81	almost perfect
Squeaky toy (final)	92	0.78	91	0.76	93	0.82	substantial
Ball toy	95	0.79	97	0.85	95	0.77	substantial
Artificial dog	95	0.86	82	0.51	83	0.56	substantial
Umbrella (initial)	95	0.85	95	0.85	93	0.8	almost perfect
Umbrella (final)	91	0.71	96	0.88	91	0.71	substantial
Commands (come)	98	0.96	95	0.88	97	0.92	almost perfect
Commands (sit)	90	0.79	84	0.67	82	0.64	substantial
Loop leash	100	1	95	0.87	95	0.87	almost perfect
AVERAGE	94.72	0.87	93.50	0.82	92.15	0.77	

The percentage of agreement (%) and Cohen’s weighted Kappa (K_w_) between three independent observers (Ob 1, Ob 2, Ob 3) for each behavioral variable outcome from both the approach and reactivity tests were calculated. Mean K_w_ values were calculated across observers and average agreement levels reported are based on Landis and Koch [32]. Explanations for each variable coding is provided in the main text.

### Approach test-retest reliability

The analysis of AT test-retest indicated that the dogs reacted similarly to the test across the three days (ICC single measure: 0.85; F = 18.58, p<0.0001; 95% CI: 0.82–0.88). The LMM showed a main effect of day (F = 14.77_(2,589)_, p = <0.0001), but not of reactivity test day (RT_day: F = 0.19_(1,589)_, p = 0.661) and an interaction effect of day*RT_day (F = 4.01_(2,589)_, p = 0.019; Table 4 for estimates of fixed effects).

**Table 4:** Linear Mixed Model output. Estimates of fixed effects (i.e., day, reactivity test day (RT_day) and their interaction) on the approach test total score (AT_tot).

Fixed Effect					95% confidence interval	
	Coefficient	Std. Error	t	p-level	lower	upper
Intercept	8.24	0.35	18.61	0.000	5.84	7.22
Day 1	-0.31	0.19	-1.69	0.09	-0.68	0.52
Day 2	-0.02	0.19	-0.12	0.90	-0.39	0.34
Day 3	-	-	-	-	-	-
RT_day 1	0.17	0.47	0.36	0.72	-0.76	1.10
RT_day 2	-	-	-	-	-	-
Day 1*RT_day 1	-0.71	0.25	-2.83	0.005	-1.21	-0.22
Day 1*RT_day 2	-	-	-	-	-	-
Day 2*RT_day 1	-0.39	0.25	-1.55	0.12	-0.89	0.10
Day 2*RT_day 2	-	-	-	-	-	-
Day 3*RT_day	-	-	-	-	-	-

- Reference categories, values not calculated

**Fig 1:**
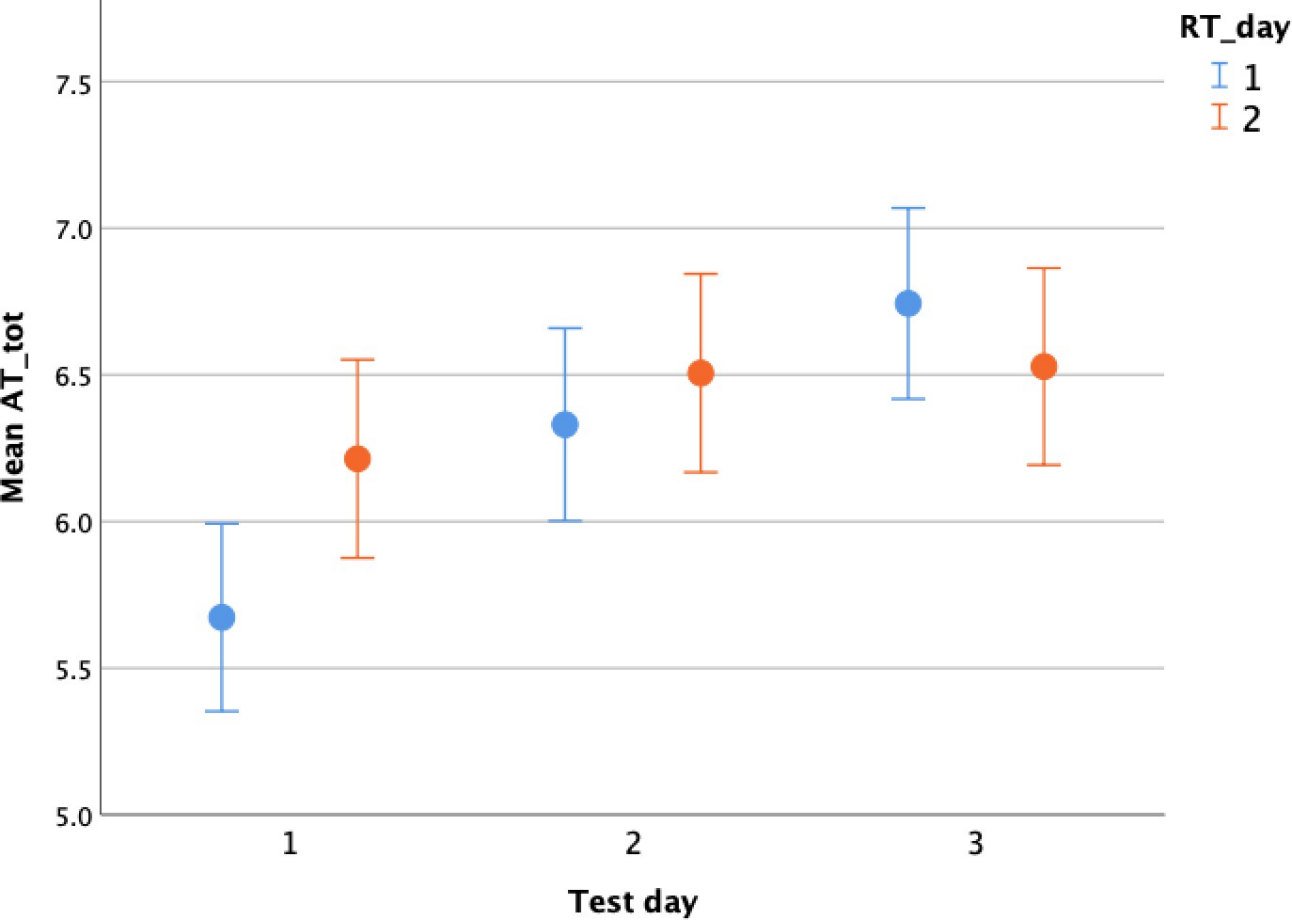
Test day and RT_day plot. Average approach test score (AT_tot: Circles and whiskers = mean and standard error bars) across the three test days with respect to the reactivity test administration day (RT_day).

Pairwise comparisons (Estimated Marginal Means) revealed an effect of RT_day only when this was administered on day 1 of testing (day 1 vs 2: t = -3.63, p = 0.001; day 1 vs 3: t = -6.08, p<0.0001; day 2 vs 3: t = -2.45, p = 0.015) but not on day 2 (day 1 vs 2: t = -1.56, p = 0.278; day 1 vs 3: t = -1.69, p = 0.278; day 2 vs 3: t = -0.12, p = 0.904). In brief, test scores (AT_tot) on days 2 and 3 improved when the RT was administered on day 1 (Fig 1).

**Fig 2:**
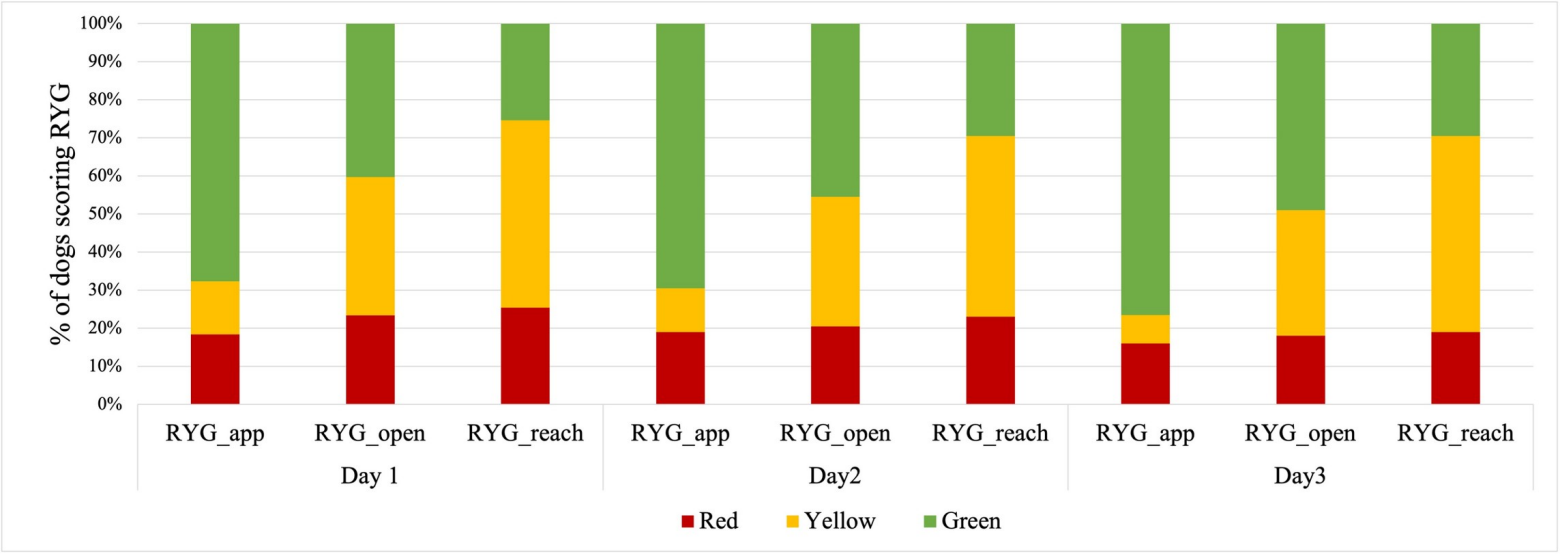
Dog response to the three phases of the approach test. Approach (RYG_app), open door (RYG_open), and reach (RYG_reach) scores using the RYG (Red, Yellow, Green) method (see main text for details) across three consecutive days.

When analyzing the dogs’ responses to the three-step AT (i.e., approach, open door, and reach), Friedman test highlighted a significant difference for all comparisons (day 1: χ^2^_(2)_ = 100.94, p<0.0001; day 2: χ^2^_(2)_ = 83.83, p<0.0001; day 3: χ^2^_(2)_ = 116.28, p<0.0001). Post-hoc pairwise comparisons using Bonferroni correction (significance level set at 0.017 for three comparisons) showed that at each step the dogs’ responses differed significantly from all other steps (p<0.0001 for all comparisons) mainly due to dogs scoring progressively worse (e.g., increased fear) as the interaction became more intense (Fig 2). This was true for all three days.

### Construct validity

The RT had to be suspended for 4 out of 447 dogs: two due to excessive fear and two due to aggressive reactions. Analysis assumptions to perform the EFA were met: communalities > 0.40 for all variables, KMO = 0.95, and a Bartlett test of Sphericity was significant (p<0.0001). The EFA extracted four main factors (Table 5) with Eigenvalue >1 (Section 6.2 in S1 File for scree plot), explaining 63.4% of total variance.

**Table 5:** Dimensions extracted by the Exploratory Factor Analysis (EFA).

Variables	Component			
	Food Motivation	Sociability	Boldness	Responsiveness
Commands (sit)/treat	**.777**	.260	.175	.348
Commands (come)/treat	**.741**	.335	.237	.249
Mat/treat	**.688**	.147	.377	.077
Commands (sit)	**.680**	.397	.259	.332
Loop leash/treat	**.648**	.206	.155	.448
RYG_reach/treat	**.646**	**.515**	.138	.113
Leash/treat	**.634**	.014	.424	.144
RYG_open/treat	**.620**	**.504**	.170	.091
RYG_app/treat	**.553**	.278	.276	.005
RYG_open	.218	**.741**	.304	.295
RYG_reach	.245	**.703**	.268	.326
TOUCH	.270	**.680**	.139	.211
RYG_app	.263	**.563**	.372	.209
Commands (come)	.387	**.531**	.350	.435
Squeaky toy (initial)	.298	.440	.386	.386
Squeaky toy (final)	.321	.420	.364	.379
Leash	.359	.275	**.720**	.221
Mat	.247	.270	**.656**	.194
Ball toy	.288	.183	**.556**	.075
Artificial dog	.124	.186	**.554**	.328
Cone	.205	.322	**.540**	.424
Problem solving	.396	.128	.407	.191
Umbrella (initial)	.160	.214	.221	**.719**
Umbrella (final)	.138	.213	.164	**.673**
Loop leash	.266	.441	.326	**.546**
Explained Variance (%)	20.8	16.4	14.2	12.0

Loadings higher than 0.50 are in bold.

All scores involving the offering of a treat (during both the AT and RT) grouped together with high loadings on the first factor, explaining 21% of the total variance. The command ‘sit’ also loaded there, which was foreseeable since the dog was lured to a sit position with a treat. This factor was labelled Food Motivation. The RYG scoring from all three steps of the AT and whether the dog allowed the experimenter to touch him/her, and the score from the ‘come’ command loaded together on the second factor. The reaction to the squeaky toy had its highest loadings on this factor too, although it was not high enough to make the cut-off of 0.50. The offering of a treat during more ‘invasive’ steps of the AT such as the open and reach also cross-loaded on this factor. This component explained 16% of the variance and was labelled Sociability. The dogs’ scores in response to the leash, mat, cone, and artificial dog, loaded together on the third component. The upside-down bowl from the problem solving had its highest loadings on this factor too, although not high enough to make the cut-off of 0.50. This component explained 14% of the variance and was labelled Boldness. Finally, the last component, which explained 12% of the total variance, was labelled Responsiveness as the three variables loading here represented the reaction (initial and final) of the dogs to more socially invasive tests such as opening an umbrella and a person trying to loop a leash over their heads.

#### Effect of tester and RT_day

Results showed no effect of RT_day on all four component scores (Table 6 and Section 6.3 in S1 File for model estimates)

**Table 6:** Linear Mixed Model output. Main effect of tester and reactivity test day (RT_day) on the four main component scores extracted from the EFA.

	Food motivation		Sociability		Boldness		Responsiveness	
Fixed Factor	F(df)	p-value	F(df)	p-value	F(df)	p-value	F(df)	p-value
Intercept	0.02_(1,24)_	0.90	0.05_(1,24)_	0.83	0.09_(1,23)_	0.75	0.00_(1,23)_	0.96
Tester	0.03_(1,24)_	0.86	0.24_(1,24)_	0.63	1.06_(1,23)_	0.31	1.28_(1,23)_	0.27
RT_day	0.21_(1,413)_	0.64	0.07_(1,413)_	0.79	0.08_(1,412)_	0.78	0.67_(1,412)_	0.42

### Internal consistency

Internal consistency was calculated for all four factors extracted by the EFA using Cronbach’s alpha. Cronbach’s alpha values were high (>0.80) for all these factors: Food Motivation = 0.93; Sociability = 0.92; Boldness = 0.86 and Responsiveness = 0.81.

### Applied value

**Fig 3:**
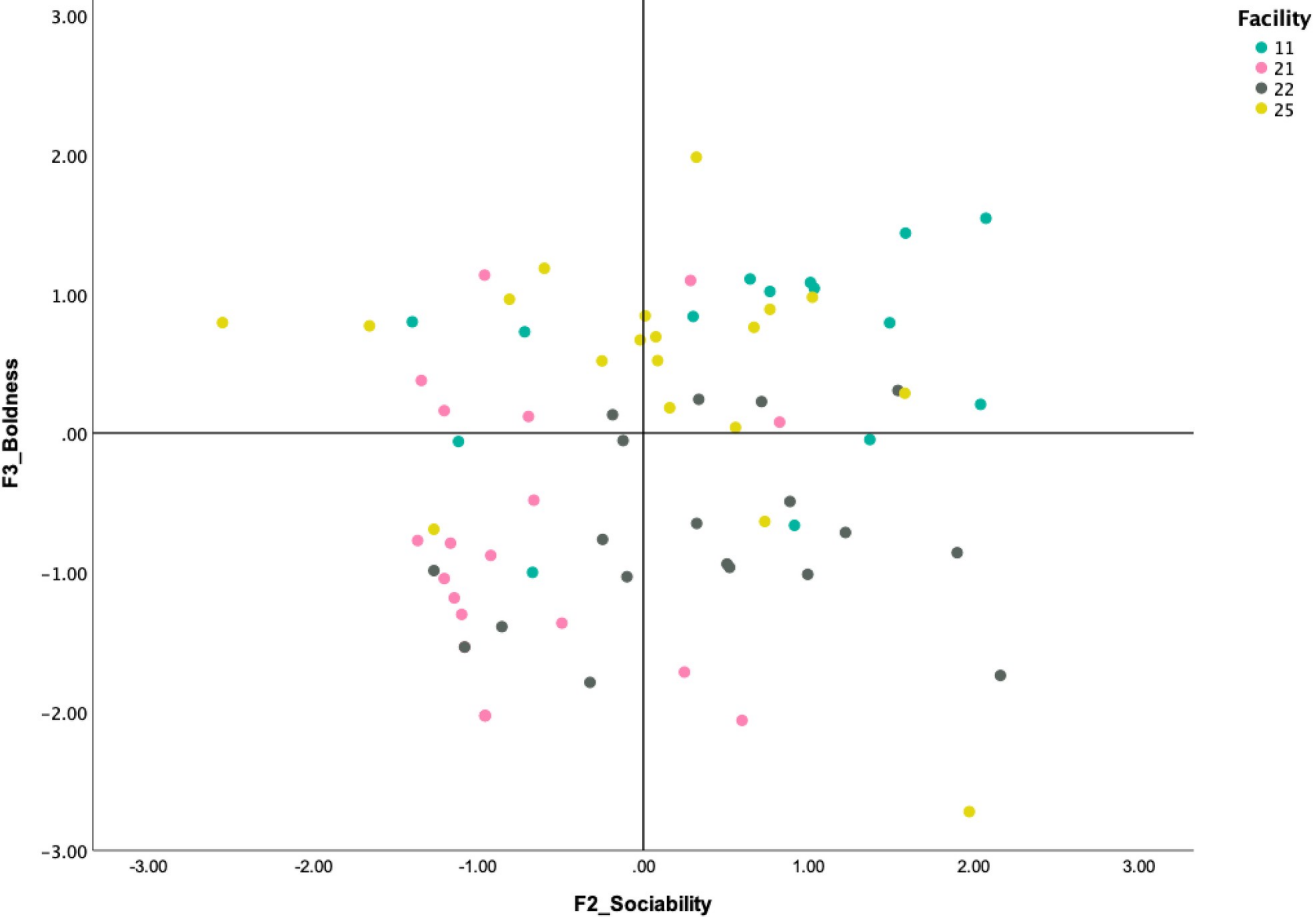
Example plot 1. Standardized factor scores (generated by the EFA) for each of the dogs tested in four of our facilities are plotted against two of the main behavioral factor scores extracted by the EFA: Sociability (F2) and Boldness (F3). Higher values on the X-axis represent scores for more engaging and affiliative behaviors towards the experimenter; higher values on the Y-axis represent scores for more engaging and exploratory behaviors towards inanimate objects.

The results of the behavioral assessment can be plotted using the factor scores created from the EFA and interpreted. Such plots can help identify facilities that might require adjustments to their behavioral management practices based on the responses of their dogs to certain aspects of testing. For example, the plot in Fig 3 shows how dogs’ scores load on two main behavioral components emerging from the EFA: Sociability toward people and Boldness. If most dogs from one facility clustered in the top right quadrant, characterized by high boldness and high sociability, it is likely these dogs reacted positively and were friendlier when presented with the novel objects or experimenter. With some exceptions, Facilities 11 and 25 (depicted in Fig 3) had many dogs responding this way. In facility 22, however, dogs appeared to be mostly sociable with people, but showed more fearful reactions to objects (note bottom right quadrant). In contrast, in facility 21, a large group of dogs clustered in the bottom left quadrant, indicating a higher frequency of fearful reactions towards both unfamiliar people and novel stimuli (Fig 3).

**Fig 4:**
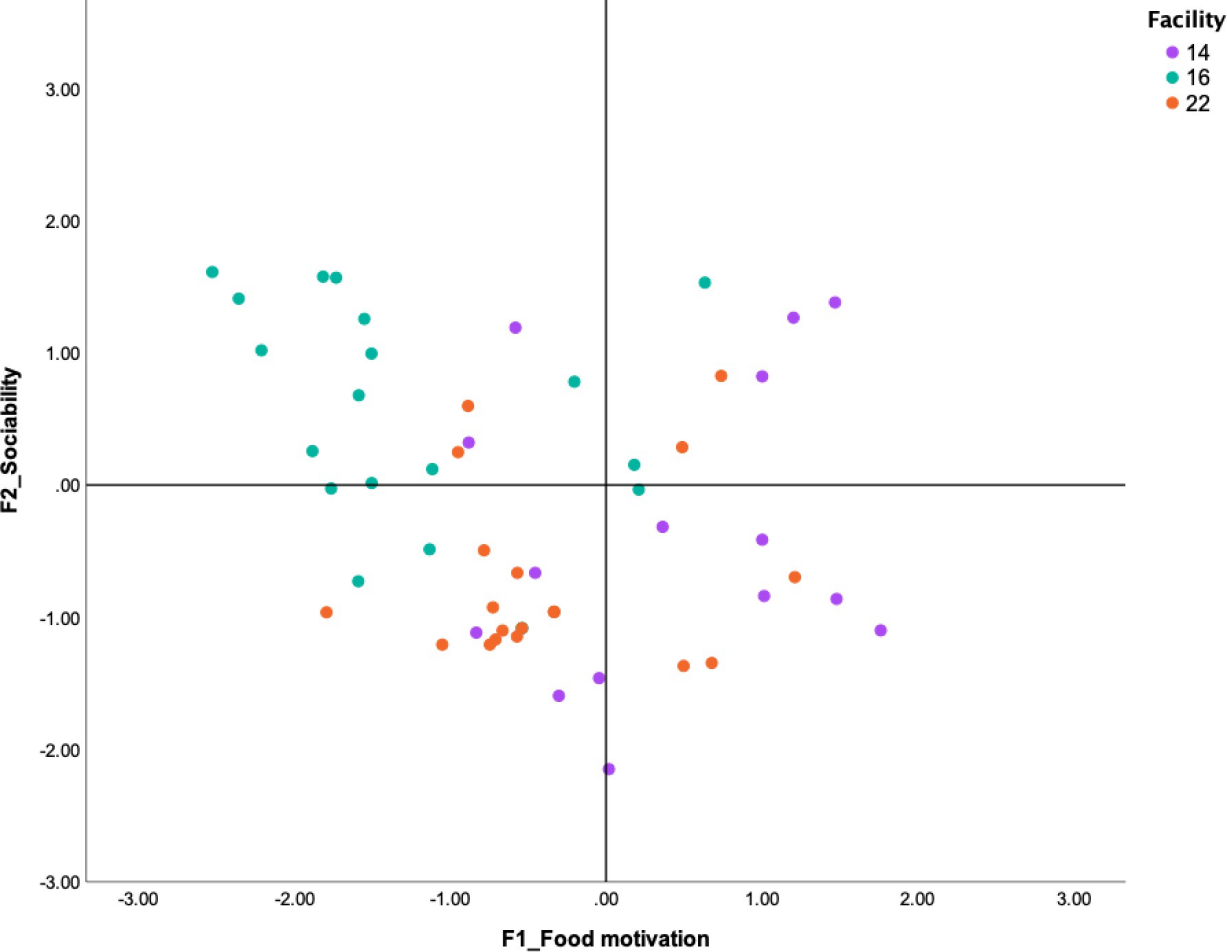
Example plot 2. Standardized factor scores (generated by the EFA) for each of the dogs tested in three of our facilities are plotted against two of the main behavioral factor scores extracted by the EFA: Food Motivation (F1) and Sociability (F2). Higher values on the X-axis represent higher propensity to take the treats offered during the test; higher values on the Y-axis represent scores for more engaging and affiliative behaviors towards the experimenter.

Fig 4 shows how dogs can be described in terms of two different components: Food Motivation and Sociability. Dogs from facility 14 loaded mainly on the bottom right quadrant and were characterized by high Food Motivation and low Sociability. Dogs in the top left quadrant (facility 16; see Fig 4) responded positively to social interactions with the experimenter even though they were not interested in the food reward. Dogs in the bottom-left quadrant did not reach to take the treats and showed fearful responses toward the experimenter.

## Discussion

Whether kenneled dogs are kept in shelters, laboratories or breeding facilities, it is important to ensure that they are provided with high standards of care that include appropriate attention to their behavioral needs. Welfare can be compromised when the animals are not offered the resources or opportunities needed for normal behavioral development, when they are not allowed to engage in behaviors that they are highly motivated to perform, or when they cannot avoid aversive situations [35]. In kennels that offer insufficient social stimulation and control over the environment, dogs may experience boredom, distress and fear, leading to states of chronic distress and abnormal behavior [36–38]. Further, an overall state of social and/or non-social fear is likely when dogs do not receive sufficient or effective socialization. This may be detrimental to the dogs’ welfare while they reside in the kennel and when introduced to new environments, especially if introduction of novel people, objects or situations cause them recurrent acute distress. Consequently, the development of a reliable tool to assess social and non-social fear in adult breeding dogs is needed to address welfare concerns linked to behavior in CB kennels.

Very little has been published about the actual behavior of dogs while they are maintained at commercial breeding kennels. Direct assessments conducted by our research group on a number of such kennels have revealed high levels of affiliative behaviors in test populations (with over 70% scoring green using FIDO testing [16, 17]) as well as moderate to high levels of social fear in some kennels (over 50% of the tested population [10]). Comprehensive behavioral assessment of breeding dogs in their kennels could contribute to changes in management practices, inform feedback provided to breeders on practices that could be implemented to reduce fear in kennels, and potentially increase the likelihood of success after rehoming. Information derived would allow development and refinement of evidence-based standards needed to promote canine welfare.

Previous research based on owner reports suggest that dogs from commercial breeding operations are more likely to show behavioral problems in a home environment after adoption than dogs from other sources [7]. How well these dogs transition to home environments after retiring from their breeding career is unknown. Some of the studies on adopted former breeding stock included dogs obtained from the puppy trade or those presumed to come from ‘puppy farms’ [3]. In other studies, the origins were uncertain, and the conditions of the animals in the original kennels were unknown. Thus, outcomes of these studies may not be broadly generalizable [3, 6]. Direct observational studies and post-adoption follow-up are therefore necessary to ensure that dogs kept in commercial breeding kennels experience “a life worth living” [39] while there, and that they can easily transition into homes at the end of their breeding careers.

To ensure feasibility and generalization of test results, the protocols used in this study were tested at diverse kennels in multiple states within the U.S., which utilized different management systems. The behavioral protocol was easy to implement in a CB kennel setting. Testing dogs in their home pens considerably reduced the time and stress that may have been associated with handling and moving them to a testing arena. Overall, the AT and RT used proved to be generally reliable, in terms of consistency of scores between observers and across time, and the measures used were meaningful, both in terms of assessing behavioral characteristics of interest (as extracted by the EFA) and practicality.

### Inter-observer reliability

Inter-observer agreement was very high (>80%, average K_w_ = 0.81) for all recorded subtests. The scoring of the reaction to the dog statue produced slightly lower agreement scores, mainly due to Observer 3 scoring differently (i.e., scoring dogs as more cautious) from the other two observers. Specifically, agreement level was moderate between Observer 3 and the other two observers (Obs 3 vs. Ob 1 = 0.51; Obs 3 vs. Obs 2 = 0.56) compared to an almost perfect agreement between Observer 1 and 2 (0.86). The percentage of agreement, however, was over 82%, so well within acceptable thresholds. This specific test was more challenging to score as the reaction of dogs to a realistic representation of a conspecific was complex, at times shifting from affiliative to fearful within a short fraction of the exploratory period. Thus, standardized scoring on this item requires more practice. To that end, Observers 1 and 2 were more senior researchers who developed the tool, while Observer 3, although experienced, was the first to be trained on its use. This suggests that the level of agreement can improve with training and practice. Keeping these concerns in mind, all live scoring included in subsequent analyses were carried out by Observers 1 and 2 to avoid compromising the reliability of the test scoring. This protocol was designed primarily to allow scientific investigations, although it has potential for future applications as stated later in the discussion. A simple 3-point scale scoring system was selected to facilitate standardization, however, it is important to note that the reliability of this scoring system was tested only among well-trained researchers familiar with dog behavior, so any future applications would have to be supported by further context-specific reliability studies.

### Test-retest reliability

Results from the AT indicated that the dogs’ responses to the experimenter approaching the pen were highly correlated across the three days of testing. However, dogs scored significantly better on days 2 and 3, but only if they received the RT on the first day. It could be argued that the administration of the RT on day 1, which involved the (presumably) positive experience of gentle interactions with the researchers and treats, increased the likelihood of dogs being more affiliative on the following days. Why RT given on day 2 did not have the same significant positive effect on day 3 AT is unclear. However, the plot (Fig 1) shows that the group of dogs that received the RT on day 1 scored lower on average than the other group on the AT_tot score. Although the difference between the two groups on day 1 was not significant, the dogs that scored lower on AT_tot had a higher behavioral variation, scoring similarly to the other group by day 3. In conclusion, this could be a sample effect (i.e., this effect may disappear by increasing the sample size). Interestingly, and in support of this, there was no RT_day effect in the later analysis which included a larger sample size and both AT and RT scores (Table 6).

Test-retest analysis and its limitations in the canine behavior literature has been challenged before [29, 40]. In fact, no consensus has been reached on the amount of time that should pass between tests to confirm repeatability (i.e., that the responses to the test are consistent across time), the effect of learning on dogs’ responses when experiencing the same test multiple times, or the minimum level of agreement that is acceptable between consecutive scores to warrant trait stability. In our study, test-retest was performed on three consecutive days. An effect of repetition was expected as dogs became familiarized with the same experimenter during the study. The analysis revealed that although some dogs improved their average scores, overall the type of response remained consistent, meaning that a fearful dog was unlikely to become non-fearful of an unfamiliar person even after those limited repeated exposures. In terms of test applicability, it appears that, for this dog population, performing the AT is a quick and easy way to provide a reliable indication of the level of fearfulness dogs toward strangers at an individual or kennel level.

Previous work on the FIDO evaluation tool, and the approach test portion in particular, demonstrated that only the initial reaction of dogs to the test could be scored without losing important information [10, 17]. On the basis of those results, a revised three-step protocol based on initial dog reactions was developed. In the current study, it was important to confirm whether these three steps elicited a different response in dogs (as observed in [10]) (i.e., at every step the level of interaction progressively intensified, likely eliciting higher levels of fear). This suggestion is bolstered by findings when comparing the dogs’ responses at each step (see Fig 2). As the test progressed, the proportion of affiliative reactions (indicated in green) decreased whereas ambivalent reactions (shown in yellow) increased. This observation has important implications for assessing the sociability of a dog, especially in relation to their suitability for rehoming, where direct contact and interactions with unfamiliar people may be more common and unavoidable. The AT simulates an interaction that is foreseeable once the dog is rehomed. Thus, it may help to identify those individuals that are less likely to cope well with novel human interactions that may take place after rehoming. It is important to mention that our preliminary observations (unpublished data) suggest that some dogs from commercial kennels that react fearfully to strangers are highly affiliative toward their own caretakers, underscoring the need for breeders to implement effective socialization protocols with unfamiliar people to help dogs generalize positive human interactions to strangers.

### Construct validity

The EFA analyses of our data extracted four main components, the first loading all the variables related to receiving a treat from the researcher. This factor is interesting, as it indicates that level of food motivation may affect a dog’s response to the test. Highly food motivated dogs may have overcome their fear and attempted to reach for the hand with the treat. For these dogs, it is possible that food may be used effectively for behavioral modification to help reduce fear. Since food can be a strong reinforcer for shaping behavior, its use may help dogs cope with transitioning to homes and other new environments. In contrast, characterizing a dog on two behavioral components (Food motivation and Sociability) may facilitate identifying and strategizing on alternative approaches if a dog is not food motivated and is also fearful. There may be dogs, for example, that are people-oriented for which food may not be as high value (e.g., see Fig 4, facility 14). Such dogs seek human attention, and therefore, social contact or touch could be used to reinforce desired behaviors instead of food. In addition, such dogs may rely on their new owners as stress buffers when transitioning to their new homes [41]. Some dogs in our sample were characterized as low on both Food Motivation and Sociability (e.g., see Fig 4, facility 22). Even if these dogs were simply too fearful of the experimenters to eat during testing, it is plausible that they may have more difficulties adapting to a new family environment, with unfamiliar people around, especially since food may be a less effective reinforcer for them.

The second EFA component was Sociability. Variables related to the reaction of dogs during the approach test (AT), including when offering treats during the open and reach steps, and during the ‘come’ command, all loaded on this component. The ‘come’ command was expected to load under Sociability, since it involved the researchers opening the pen door and actively inviting the dog to come within reach, hence, it was a measure of sociability similar to the ‘reach’ phase of the AT. The squeaky toy variables, even if not strongly, loaded highly on this factor too and are worth mentioning. The toy was the only object manipulated in close proximity to the dog by the researcher and with no visual block between them (as was the case for the umbrella), whereas all other objects were placed inside the pen and left by the researcher after closing the door and stepping back. Although the squeaky toy was intended to measure the reaction to the sound of a common dog toy, this loading suggests that the proximity of the researcher may have been the major stimulus for these dogs’ responses, rather than the novelty or sound of the toy.

The third component was labeled Boldness. During the RT, dogs were exposed to a range of novel stimuli with each being introduced to measure different traits. Although a range of responses was recorded for these stimuli, indicating behavioral variability in this population of dogs, the EFA suggests that the novelty of the test situation and also of each object elicited similar levels of avoidance or exploration depending on the overall level of fearfulness or boldness of the dog. Previous studies with shelter dogs have demonstrated that this behavioral test is a robust measure of traits such as sociability to humans, sociability to conspecifics, and playfulness [20, 21]. In this population of dogs, the sociability to humans component was clearly maintained, whereas playfulness and sociability to conspecifics were not detected by this assessment. The playfulness trait is normally characterized by the dogs’ responses to the squeaky toy and ball [20, 21]. It is possible that many of the subject dogs had not been exposed to these kinds of commonly used dog toys and therefore they did not elicit play behavior. As these two variables did not measure playfulness, they may instead have been re-distributed by the analysis on two different factors (i.e., Sociability and Boldness). The failure to detect a component describing attitude towards conspecifics may be due to the refinement and adaptations of the test. Previous studies have shown that an artificial dog can be used to elicit social reactions resembling those directed to other dogs [20, 42, 43]. In the present study, the presentation of the artificial dog was used as a proxy measure of dogs’ sociability, but the EFA revealed that this stimulus was responded to similarly to other novel objects presented. However, exploratory analysis using a more refined ethogram suggests that the dogs were actually reacting significantly differently to the artificial dog than, for example, the cone, in terms of types and frequencies of behaviors displayed [44]. When interacting with the artificial dog, our subjects showed less fear and more social behaviors (i.e., behaviors shown when interacting with a conspecific such as approaching with a low or a high body posture, increased duration of sniffing) than when introduced to the plastic cone [44]. It is possible that the scoring system used here was unable to capture qualitative differences. Therefore, additional studies need to be conducted to more accurately understand how dogs from CB kennels perceive these objects.

The final factor extracted by the EFA was labelled Responsiveness. The umbrella represented a typical startle test [45, 46], eliciting an initial avoidance response that faded after a few repetitions, as the dog habituated to the harmless stimulus. The other variable loading on Responsiveness was the reaction to the leash being looped over the dog’s head. Many dogs from CB kennels are not leash trained and have never worn a leash. Although the leash was left inside the pen for the entire duration of the RT in order to familiarize the dog with it and reduce its novelty, dogs still responded fearfully to the sight of the leash being manipulated by the experimenter. It is important to remember that to protect the dogs from any unnecessary stress, they were never cornered or forced to wear the leash. In addition, the dog was free to approach and retreat from it at all times. Interestingly, as reported in Barnard et al. [20], these variables (reaction to the opening of an umbrella and placing on leash) loaded on the same single component, suggesting that for dogs that are unused to wearing a collar or leash, this action may be perceived as particularly aversive, especially if the subject lacks socialization and is afraid of the unfamiliar person manipulating the leash.

Finally, we urge caution when interpreting the word “reactivity”. In non-scientific settings, this term is often used colloquially as a synonym for aggression. In the present study (see introduction), the definition of reactivity is broader and not limited to aggressive responses. In fact, the prevalence of aggressive reactions in this study was very low: only 19/447 dogs (4.3%) showed signs of aggression at least once during the assessment, and for just 2/447 dogs, the assessment had to be ended due to aggressive behavior.

Although acknowledging the limitation of using EFA with categorical variables, this analysis should be taken as an exploratory exercise that provided a good understanding of the association between individual variables and the latent behavioral traits that we were likely measuring when administering this test to this population of dogs. It is worth mentioning that some authors have challenged the purely statistical concept of test validity as it is often presented, in favor of a more theoretical and ontological approach [47]. In other words, validity is not something that is investigated after the test has been applied; rather it happens at the construction stage of the test [47]. Construct validity can be defined as the ability of a test score to convey the variation in the attribute it intends to measure. This is possible only if during conception, test scores were generated on the basis of the hypothesis that there is a causal association between variation in the attribute (e.g., fearfulness) and the measurement outcome (e.g., RYG scoring). Based on our initial effort at the time of test development and on the results presented here, we contend that this test largely measured what it was designed to measure, hence providing support for construct validity at both statistical and theoretical levels.

#### Effect of tester and RT_day

The analysis of the entire behavioral assessment (i.e., including both the AT and RT) showed that there was no effect of the tester, meaning that different trained researchers could perform the tests in a standard manner without affecting the dogs’ response. It is important to point out, however, that for this study, two researchers (both Caucasian and female) who were initially trained together performed the tests. In any future research using these assessment tools, potential effects between experimenters conducting the tests be evaluated, particularly if the testers are of different ethnicities, races, or genders, as there has been evidence in some cases of dogs reacting differently to female or male testers [48].

### Internal consistency

The high internal consistency calculated for each of the four factors confirmed that the variables loading on the same component were measuring the same construct [28]. Although the behavioral assessment (including habituation time to isolation) lasted around 15 minutes per dog, which makes this protocol very easy to implement, results from the EFA and internal consistency analyses suggest that some of the items within this assessment may be redundant (e.g., the RT ‘come’ command and the AT ‘Reach’ step). An additional refinement of the test that eliminates redundant items could reduce testing time without compromising the quality and variety of behavioral welfare information.

### Applied value

The current assessment tool has strong potential for application, as it may help breeders identify the prevalence of social and non-social fear in their kennels and inform their overall socialization and other behavioral management practices. In addition, the assessment tool could provide insights into implementing individual dog behavior modification plans for animals at higher risk of experiencing chronic states of fear and stress. Both cartesian plots (see Figs 3 and 4) showed that there was behavioral variability within each facility. The graphs also showed how these results may generate information on dogs’ levels of neophobia and sociability, which in turn might help to predict how they may cope with novelty in the kennel or when transitioning to a new home. Such information can be used to improve the socialization and welfare outcomes of dogs in a specific kennel. For example, based on the findings, facility 22 (see Fig 3) may have been appropriately socializing their dogs to people, but insufficiently exposing the dogs to novel objects to reduce their levels of neophobia. In another example, the high prevalence of dogs from facility 21 (see Fig 3) showing fearful behavior towards both the experimenter and objects suggests that there may be potential for improvement in all of that kennel’s socialization practices, in order to attenuate fear in the dogs. In these examples, the information generated from the assessment tool could be used to design specific or generalized interventions and evaluate their effects. For example, if only a few dogs showed high levels of fear, breeders could work specifically with more fearful individuals, especially for potential rehoming. In other cases, if most of the dogs in the facility showed signs of fear, whether toward unfamiliar people or objects, breeders could work to implement new breeding selection, management and socialization practices that could benefit not just the most vulnerable dogs, but the entire kennel population. The behavioral assessment tool presented here can be implemented on its own to focus on the prevalence of fear in CB kennels, or it can be integrated into more comprehensive protocols (including e.g., physical, physiological metrics) for overall welfare assessments.

It is important to acknowledge that although we had a wide range of kennels that used a variety of management systems, as is the case for our previous studies [8–10, 16, 17], all breeders voluntarily participated in the current study. This may have introduced a skew in the sample toward breeders who were already operating at a higher standard than many of their peers, and were therefore unconcerned about permitting studies to be conducted on their premises. Despite this caveat, it is important that scientists avoid making or repeating assumptions that commercial breeding kennels are one and the same as ‘puppy mills’ or that US commercial breeders offer uniformly poor care and welfare standards. Particularly since much of the basis for such views has been derived indirectly and in light of emerging scientific and empirical evidence contradicting such beliefs, it is essential to retain objectivity to avoid both overly positive and negative characterizations of the industry that inaccurately represent it and potentially complicate welfare assessment and improvement of conditions at commercial breeding operations.

Building on the current findings, in future studies, it will be important to collect data from a larger sample that includes an even wider range of breeding and management systems. Such studies should also evaluate which management practices may affect (both positively and negatively) the behavior and welfare of dogs in commercial breeding kennels and determine how these conditions affect dogs’ abilities to transition to new homes after retirement from breeding.

## Conclusions

The behavioral assessment tool used in the current study was found to have reliable measures that scored consistently across different observers and across time. Construct validity and internal consistency were demonstrated, barring a few limitations, and the behavioral assessment was determined to be practical and meaningful for use with this population of dogs. It was interesting to note that this battery of tests appeared to primarily measure fear of novelty (whether toward people, stationary or moving objects) in this population of dogs, rather than a more complex range of behavioral and cognitive traits that have emerged when testing companion dogs in homes or shelter populations. Indeed, the simplified adaptation of these tests may have affected the results. Alternatively, it is possible that many dogs in the tested kennels had limited or no exposure to objects like bowls, leads, or balls, resulting in constraints in their ability to demonstrate traits such as playfulness, problem-solving ability, trainability, etc. when presented with them.

Regardless, this test appears to offer a useful screening tool for assessing the levels of social and non-social fear towards people and objects exhibited by dogs in commercial breeding populations. Incorporating the test into comprehensive canine welfare assessments could help to identify individual dogs within kennels that may be experiencing unnecessary fear and/or distress when faced with novel objects or unfamiliar people, which could result in compromised welfare. Targeted behavioral interventions for these individuals could then be developed to improve their quality of life while in the kennel that may also be beneficial in helping them to adapt to life outside of the kennel. Additionally, the screening tool could be useful in identifying key areas for improvement in behavioral management for all dogs in a CB kennel. This could be an important step in addressing broader welfare concerns associated with the behavioral wellness of dogs originating from such kennels and also aid in setting dogs up for success when rehomed at the end of their breeding careers. Ultimately, while post-adoption assessments would be needed to understand how these dogs transition out of CB kennels to homes, this screening tool may permit benchmarking of behaviors that can provide a basis for predicting rehoming outcomes and preparing dogs to transition to homes.

## Supporting information

S1 File This document contains additional information and detail descriptions of the pilot testing performed and the tests protocols used in this study. (DOCX)

## References

[1] Croney CC. Turning up the volume on man’s best friend: ethical issues associated with commercial dog breeding. J Appl Anim Ethics Res. 2019;1: 230–253. doi: 10.1163/25889567-12340011

[2] McMillan FD. Behavioral and psychological outcomes for dogs sold as puppies through pet stores and/or born in commercial breeding establishments: Current knowledge and putative causes. J Vet Behav Clin Appl Res. 2017;19: 14–26. doi: 10.1016/j.jveb.2017.01.001

[3] Wauthier LM, Scottish Society for the Prevention of Cruelty to Animals (Scottish SPCA), Williams JM. Using the mini C-BARQ to investigate the effects of puppy farming on dog behaviour. Appl Anim Behav Sci. 2018;206: 75–86. doi: 10.1016/j.applanim.2018.05.024

[4] Bir C, Widmar NJO, Croney CC. Stated Preferences for Dog Characteristics and Sources of Acquisition. Animals. 2017; 7(8): 59. 10.3390/ani7080059. PMC5575571 28783072

[5] Bir C, Widmar NJO, Croney CC. Exploring Social Desirability Bias in Perceptions of Dog Adoption: All’s Well that Ends Well? Or Does the Method of Adoption Matter?. Animals. 2018; 8(9): 154. doi: 10.3390/ani8090154 30217039 PMC6162534

[6] McMillan FD, Serpell JA, Duffy DL, Masaoud E, Dohoo IR. Differences in behavioral characteristics between dogs obtained as puppies from pet stores and those obtained from noncommercial breeders. J Am Vet Med Assoc. 2013;242: 1359–1363. doi: 10.2460/javma.242.10.1359 23634679

[7] McMillan FD, Duffy DL, Serpell JA. Mental health of dogs formerly used as “breeding stock” in commercial breeding establishments. Appl Anim Behav Sci. 2011;135: 86–94. doi: 10.1016/j.applanim.2011.09.006

[8] Stella J, Hurt M, Bauer A, Gomes P, Ruple A, Beck A, et al. Does flooring substrate impact kennel and dog cleanliness in commercial breeding facilities? Animals. 2018;8: 59. doi: 10.3390/ani8040059 29690514 PMC5946143

[9] Stella J, Bauer A, Croney C. A cross-sectional study to estimate prevalence of periodontal disease in a population of dogs (Canis familiaris) in commercial breeding facilities in Indiana and Illinois. PLoS One. 2018;13. doi: 10.1371/journal.pone.0191395 29346448 PMC5773197

[10] Stella J, Shreyer T, Ha J, Croney C. Improving canine welfare in commercial breeding (CB) operations: Evaluating rehoming candidates. Appl Anim Behav Sci. 2019;220: 104861. doi: 10.1016/j.applanim.2019.104861

[11] Beerda B, Schilder MBH, Van Hooff J, De Vries HW, Mol JA. Chronic stress in dogs subjected to social and spatial restriction. I. Behavioral responses. Physiol Behav. 1999;66: 233–242. doi: 10.1016/s0031-9384(98)00289-3 10336149

[12] Beerda B, Schilder MBH, Bernadina W, Van Hooff J, De Vries HW, Mol JA. Chronic stress in dogs subjected to social and spatial restriction. II. Hormonal and immunological responses. Physiol Behav. 1999;66: 243–254. doi: 10.1016/s0031-9384(98)00290-x 10336150

[13] Hetts S, Derrell Clark J, Calpin JP, Arnold CE, Mateo JM. Influence of housing conditions on beagle behaviour. Appl Anim Behav Sci. 1992;34: 137–155. doi: 10.1016/S0168-1591(05)80063-2

[14] Rooney NJ, Gaines SA, Bradshaw JWS. Behavioural and glucocorticoid responses of dogs (Canis familiaris) to kennelling: Investigating mitigation of stress by prior habituation. Physiol Behav. 2007;92: 847–854. doi: 10.1016/j.physbeh.2007.06.011 17617429

[15] Hiby EF, Rooney NJ, Bradshaw JWS. Behavioural and physiological responses of dogs entering re-homing kennels. Physiol Behav. 2006;89: 385–391. doi: 10.1016/j.physbeh.2006.07.012 16905163

[16] Bauer AE, Jordan M, Colon M, Shreyer T, Croney CC. Evaluating FIDO: Developing and pilot testing the Field Instantaneous Dog Observation tool. Pet Behav Sci. 2017;4: 1. doi: 10.21071/pbs.v0i4.5766

[17] Mugenda L, Shreyer T, Croney C. Refining canine welfare assessment in kennels: evaluating the reliability of Field Instantaneous Dog Observation (FIDO) scoring. Appl Anim Behav Sci. 2019;221: 104874. doi: 10.1016/j.applanim.2019.104874

[18] Jones AC, Gosling SD. Temperament and personality in dogs (Canis familiaris): A review and evaluation of past research. Appl Anim Behav Sci. 2005;95: 1–53. doi: 10.1016/j.applanim.2005.04.008

[19] Sforzini E, Michelazzi M, Spada E, Ricci C, Carenzi C, Milani S, et al. Evaluation of young and adult dogs’ reactivity. J Vet Behav Clin Appl Res. 2009;4: 3–10. doi: 10.1016/j.jveb.2008.09.035

[20] Barnard S, Kennedy D, Watson R, Valsecchi P, Arnott G. Revisiting a previously validated temperament test in shelter dogs, including an examination of the use of fake model dogs to assess conspecific sociability. Animals. 2019;9: 835. doi: 10.3390/ani9100835 31635203 PMC6826718

[21] Valsecchi P, Barnard S, Stefanini C, Normando S. Temperament test for re-homed dogs validated through direct behavioral observation in shelter and home environment. J Vet Behav Clin Appl Res. 2011;6: 161–177. doi: 10.1016/j.jveb.2011.01.002

[22] Fraser D, Weary DM, Pajor EA, Milligan BN. A scientific conception of animal welfare that reflects ethical concerns. Animal Welfare. 1997; 6:187–205.

[23] Foyer P, Svedberg AM, Nilsson E, Wilsson E, Faresjö Å, Jensen P. Behavior and cortisol responses of dogs evaluated in a standardized temperament test for military working dogs. J Vet Behav Clin Appl Res. 2016;11: 7–12. doi: 10.1016/j.jveb.2015.09.006

[24] Shyness Svartberg K. and boldness predicts performance in working dogs. Appl Anim Behav Sci. 2002;79: 157–174.

[25] van der Borg JAM, Netto WJ, Planta DJU. Behavioural testing of dogs in animal shelters to predict problem behaviour. Appl Anim Behav Sci. 1991;32: 237–251. doi: 10.1016/S0168-1591(05)80047-4

[26] Goddard ME, Beilharz RG. Early prediction of adult behaviour in potential guide dogs. Appl Anim Behav Sci. 1986;15: 247–260. doi: 10.1016/0168-1591(86)90095-X

[27] Harvey ND, Craigon PJ, Sommerville R, McMillan C, Green M, England GCW, et al. Test-retest reliability and predictive validity of a juvenile guide dog behavior test. J Vet Behav Clin Appl Res. 2016;11: 65–76. doi: 10.1016/j.jveb.2015.09.005

[28] Taylor KD, Mills DS. The development and assessment of temperament tests for adult companion dogs. J Vet Behav Clin Appl Res. 2006;1: 94–108. doi: 10.1016/j.jveb.2006.09.002

[29] Patronek GJ, Bradley J, Arps E. What is the evidence for reliability and validity of behavior evaluations for shelter dogs? A prequel to “No better than flipping a coin”. J Vet Behav Clin Appl Res. 2019; 31: 43–58. doi: 10.1016/j.jveb.2019.03.001

[30] Patronek GJ, Bradley J. No better than flipping a coin: Reconsidering canine behavior evaluations in animal shelters. J Vet Behav Clin Appl Res. 2016;15:66–77. doi: 10.1016/j.jveb.2016.08.001

[31] Cohen J. Weighted kappa: Nominal scale agreement provision for scaled disagreement or partial credit. Psychol Bull. 1968;70: 213–220. doi: 10.1037/h0026256 19673146

[32] Landis JR, Koch GG. The measurement of observer agreement for categorical data. Biometrics. 1977;33: 159. doi: 10.2307/2529310 843571

[33] Baglin J. Improving your exploratory factor analysis for ordinal data: A demonstration using FACTOR. Practical Assessment, Research, and Evaluation. 2014;19:1–15. doi: 10.7275/dsep-4220

[34] DiStefano C, Zhu M, Mîndrilã D. Understanding and Using Factor Scores: Considerations for the Applied Researcher. Practical Assessment, Research, and Evaluation. 2009;14. doi: 10.7275/da8t-4g52

[35] Dawkins MS. Behavioural deprivation: A central problem in animal welfare. Appl Anim Behav Sci. 1988;20: 209–225. doi: 10.1016/0168-1591(88)90047-0

[36] Hubrecht RC, Serpell JA, Poole TB. Correlates of pen size and housing conditions on the behavior of kenneled dogs. Appl Anim Behav Sci. 1992;34: 365–383. doi: 10.1016/S0168-1591(05)80096-6.

[37] Stephen JM, Ledger RA. An audit of behavioral indicators of poor welfare in kenneled dogs in the United Kingdom. J Appl Anim Welf Sci. 2005;8: 79–95. doi: 10.1207/s15327604jaws0802_1 16277592

[38] Taylor KD, Mills DS. The effect of the kennel environment on canine welfare: A critical review of experimental studies. Anim Welf. 2007;16: 435–447.

[39] Mellor D. Updating animal welfare thinking: moving beyond the “Five Freedoms” towards “A Life Worth Living.” Animals. 2016;6: 21. doi: 10.3390/ani6030021 27102171 PMC4810049

[40] Svartberg K, Tapper I, Temrin H, Radesäter T, Thorman S. Consistency of personality traits in dogs. Anim Behav. 2005;69(2):283–91. doi: 10.1016/j.anbehav.2004.04.011

[41] Gácsi M, Maros K, Sernkvist S, Faragó T, Miklósi Á. Human analogue safe haven effect of the owner: behavioural and heart rate response to stressful social stimuli in dogs. PLoS One. 2013;8(3):e58475. doi: 10.1371/journal.pone.0058475 23469283 PMC3587610

[42] Shabelansky A, Dowling-Guyer S, Quist H, D’Arpino SS, McCobb E. Consistency of shelter dogs’ behavior toward a fake versus real stimulus dog during a behavior evaluation. Appl Anim Behav Sci. 2015;163: 158–166. doi: 10.1016/j.applanim.2014.12.001

[43] Barnard S, Siracusa C, Reisner I, Valsecchi P, Serpell JA. Validity of model devices used to assess canine temperament in behavioral tests. Appl Anim Behav Sci. 2012;138: 79–87. doi: 10.1016/j.applanim.2012.02.017

[44] Pritchett M, Barnard S, Croney C. Socialization in commercial breeding kennels: The use of novel stimuli to measure social and non-social fear in dogs. Animals. 2021;11(3):890. doi: 10.3390/ani11030890 33804748 PMC8003938

[45] Beerda B, Schilder MBH, van Hooff J, de Vries HW, Mol JA. Behavioural, saliva cortisol and heart rate responses to different types of stimuli in dogs. Appl Anim Behav Sci. 1998;58: 365–381.

[46] King T, Hemsworth PH, Coleman GJ. Fear of novel and startling stimuli in domestic dogs. Appl Anim Behav Sci. 2003;82: 45–64. 10.1016/S0168-1591(03)00040-6.

[47] Borsboom D, Mellenbergh GJ, Van Heerden J. The concept of validity. Psychological review. 2004;111(4):1061. doi: 10.1037/0033-295X.111.4.1061 15482073

[48] Wells DL, Hepper PG. Male and female dogs respond differently to men and women. Appl Anim Behav Sci. 1999;61: 341–349. doi: 10.1016/S0168-1591(98)00202-0

