# Supplementary material for: Evaluation of an easy-to-use protocol for assessing behaviors of dogs retiring from commercial breeding kennels
Source: PLoS One. 2021 Aug 13;16(8):e0255883. doi: 10.1371/journal.pone.0255883 (PMC8362968; doi:10.1371/journal.pone.0255883)
Supplement: S1 File — (DOCX) [file pone.0255883.s001.docx]

**S1 File**

**Evaluation of an easy-to-use protocol for assessing behavioral traits of dogs retiring from commercial breeding kennels**

Shanis Barnard^¶^, Hannah Flint^¶^, Traci Shreyer, Candace Croney

Department of Comparative Pathobiology, Purdue University, West Lafayette, IN, USA

^¶^The authors contributed equally to the paper.

**Section 1: Table S1**

**Table S1. Number of subjects per breed and sex**

| **Breed** | **Female** | **Male** | **Total** |
| --- | --- | --- | --- |
| Alaskan Malamute | 3 | 2 | 5 |
| American Cocker Spaniel | 28 | 2 | 30 |
| American Eskimo Dog | 3 | 0 | 3 |
| Australian Shepherd | 12 | 3 | 15 |
| Bernese Mountain Dog | 2 | 1 | 3 |
| Bichapoo | 1 | 0 | 1 |
| Bichon Frise | 13 | 1 | 14 |
| Boston Terrier | 2 | 1 | 3 |
| Boxer | 5 | 0 | 5 |
| Bullmastiff | 9 | 1 | 10 |
| Cavalier King Charles Spaniel | 36 | 7 | 43 |
| Corgi | 3 | 1 | 4 |
| Coton de Tulear | 1 | 0 | 1 |
| French Bulldog | 15 | 3 | 18 |
| German Shepherd Dog | 1 | 0 | 1 |
| Golden Doodle | 3 | 0 | 3 |
| Golden Retriever | 18 | 4 | 22 |
| Great Dane | 11 | 5 | 16 |
| Havanese | 11 | 5 | 16 |
| Labrador Retriever | 25 | 5 | 30 |
| Lhasa Apso | 2 | 0 | 2 |
| Maltese | 6 | 3 | 9 |
| Miniature Australian Shepherd | 1 | 1 | 2 |
| Miniature Pinscher | 3 | 0 | 3 |
| Miniature Poodle | 4 | 3 | 7 |
| Miniature Schnauzer | 14 | 4 | 18 |
| Morkie | 1 | 0 | 1 |
| Neopolitan Mastiff | 7 | 2 | 9 |
| Newfoundland | 0 | 1 | 1 |
| Old English Sheepdog | 0 | 1 | 1 |
| Oripei | 1 | 0 | 1 |
| Pomeranian | 13 | 2 | 15 |
| Pomsky | 0 | 1 | 1 |
| Pug | 4 | 0 | 4 |
| Rottweiler | 4 | 1 | 5 |
| Saint Bernard | 7 | 2 | 9 |
| Samoyed | 2 | 0 | 2 |
| Shetland Sheepdog | 13 | 1 | 14 |
| Shiba Inu | 5 | 1 | 6 |
| Shih Tzu | 22 | 5 | 27 |
| Siberian Husky | 22 | 5 | 27 |
| Standard Poodle | 5 | 1 | 6 |
| Toy Australian Shepherd | 2 | 0 | 2 |
| Toy Poodle | 11 | 8 | 19 |
| Yorkshire Terrier | 9 | 4 | 13 |
| **Total** | **360** | **87** | **447** |

**Section 2: Pilot study**

A pilot research study from our group demonstrated that the FIDO score of dogs from commercial breeding (CB) kennels while in their home pens was consistent with avoidance of an unfamiliar person in an outdoor arena (Mugenda, 2018). The current protocol was designed to be carried out in the dogs’ home pens to avoid additional stress due to handling and moving them to a different testing location. However, previous studies did not isolate the dog inside, so a new pilot study was performed to confirm that the dog’s response to an approach test while closed inside with their group of pen-mates did not differ significantly from when they were separated from them. Since closing the dogs indoors for the purpose of the test might have affected test results due to group housed dogs not being used to isolation, we carried out a pilot study on a separate group of 60 dogs and performed the approach (step 1) twice: the first time a focal dog was RYG (Red-Yellow-Green) scored while closed inside with all the pen-mates, the second time the same dog was re-scored while closed inside separated from the group. Dogs were given 3 minutes to acclimate between tests. This test was repeated over three consecutive days. Scores were summed over the three days to create one group RYG score, and one individual RYG score. Analysis highlighted a significant positive correlation between the two scores (r=0.831, p<0.0001); mean scores were slightly lower (i.e., more fearful) when isolated (4.12 vs 4.63) but this difference was not significant (p>0.05). Results suggested that dogs could be isolated inside for assessment without significantly altering their behavioral response to the test.

Mugenda L.W. 2018 *Refining and validating an on-site canine welfare assessment tool developed for use in commercial breeding kennels*. Master’s Thesis. Purdue University.

**Section 3: Approach test (AT) detailed protocol**

Conduct with the dog closed in the indoor portion of the pen. Experimenter will conduct all steps with research assistant standing out of sight and recording all scores (given verbally by experimenter). Score the dogs’ behavioral reaction according to the Red-Yellow-Green (RYG) system described in the main paper (Table 1).

In addition, note the following behaviors separately and record them only once no matter when they are observed in the AT.

| Score | Description |
| --- | --- |
| Yes= 0  No= 1 | *Aggression:* Record whether the dog shows any aggressive behavior during the test (Y/N) |
| Yes= 0  No= 1 | *Frantic/ overstimulated:* Record whether the dog is overly aroused by the presence of the investigator and struggles to calm down (Y/N) |
| Yes= 0  No= 1 | *Stereotypic behaviors:* Record whether the dog is showing repetitive behaviors such as circling, pacing or wall bouncing (Y/N) |

*Note: In the present study, these behaviors were rarely recorded so they were not included in the analysis.*

**STEP 1: Approach**

| Score | Description |
| --- | --- |
| RYG_app:  Red= 0  Yellow= 1  Green= 2  RYG_app/treat:  Yes= 1  No= 0 | - Approach the pen door and stand quietly approximately 0.5 meters from the kennel door, turned to the side, avoiding direct eye contact - Record the dog’s immediate response to approach using RYG - Wait 3 seconds, then toss a treat to the dog over the kennel door, maintaining side orientation and avoiding direct eye contact - Wait 3 seconds and record whether the dog eats the treat during the allotted time (Y/N) |

**STEP 2: Open door**

| Score | Description |
| --- | --- |
| RYG_open:  Red=0  Yellow= 1  Green= 2  RYG_open/treat:  Yes= 1  No= 0 | - Open the door of the pen and stand with side facing the dog, avoiding eye contact, and with arm slightly extended, palm up and facing the dog at approximately the dog’s head height (if needed, crouch down or bend knees) - Record the immediate response of the dog using RYG - Wait 3 seconds, then offer a treat directly from hand (hand not to be extended more than 0.5 meters from the body) to the dog while maintaining the same orientation - Wait 3 seconds and record whether the dog eats the treat during the allotted time (Y/N) - If the dog does not take the treat from the hand, then toss the treat to the dog |

**STEP 3: Reach**

| Score | Description |
| --- | --- |
| RYG_reach:  Red=0  Yellow= 1  Green= 2  TOUCH:  Yes= 1  No= 0  RYG_reach/treat:  Yes= 1  No= 0 | - While maintaining sideways orientation, reach out with hand furthest from the dog and offer a treat while simultaneously slowly extending the closer hand (not more than 0.5 m) making sure that the dog is aware of the hand reaching out, to touch the side of the dog (so the ‘touch’ is a gentle stroke on the shoulder) - Record the immediate response of the dog using RYG - Wait 3 seconds and record whether the dog allows the experimenter to touch her/him (Y/N) and whether the dog eats the treat from the hand during the allotted time (Y/N) - If dog does not take the treat from the hand, then toss the treat on the floor |

**Section 4: Reactivity test (RT) detailed protocol**

1. All tests conducted by the same experimenter, with research assistant (RA) standing out of sight and recording all scores (given verbally by the experimenter).
2. Although the test is scored live, it is recorded using a video camera for later reliability analysis.
3. If at any point signs of extreme fear or aggression that risk the safety of either the dog or experimenter are seen, then testing will be terminated.
4. For subtests scored for 30 seconds (1, 2, 3 & 6), if the dog shows behaviors from multiple categories, score the category most reflective of their behavior throughout the 30 second observation period. For subtest 7 (artificial dog) score the dog’s initial reaction during the first 10 to 15 seconds.
5. If the dog is showing stereotypic behavior and does not alter the behavior or engage with the stimuli during the subtest, score as a 0.
6. Start scoring behavior from when the door closes (for relevant items).

| **Subtest label** | **Tester behavior** | **Scoring description** | **Score** |
| --- | --- | --- | --- |
| 1. **Introduce mat**   (30 seconds) | Calmly place a thin rubber mat on the floor in front of the gate and place a treat on top. Close door and step back away from the front of the pen.  Leave mat on the floor for the rest of the test. All subsequent objects and treats will be placed on top of the mat. The mat prevents the treats from falling through the pen floor when it is perforated. | Interaction with confident/neutral posture OR ignores but steps on it with confident posture (no signs of avoidance) | 2 |
|  |  | Approach/avoidance OR exploration with lowered posture OR ignores and does not step on with confident/neutral posture | 1 |
|  |  | Afraid/avoid, increase distance AND no approach or very brief interaction with lowered posture | 0 |
|  |  | Eats treat | 1 |
|  |  | Does not eat treat | 0 |
| 1. **Introduce leash** (30 seconds) | Calmly show a slip leash to the dog and place it in a loop on the mat (to one side) with a treat on top. Close door and step back away from the front of the pen. Record reaction.  Leave leash on the floor for the rest of the test and place all subsequent objects and treats on the mat, next to the leash. | Interaction with confident/neutral posture OR ignores but stands near it with confident/neutral posture (no signs of avoidance) | 2 |
|  |  | Approach/avoidance OR exploration with lowered posture OR ignores and does not come near with confident/neutral posture | 1 |
|  |  | Afraid/avoid, increase distance AND no approach or very brief interaction with lowered posture | 0 |
|  |  | Eats treat | 1 |
|  |  | Does not eat treat | 0 |
| 1. **Cone**   (30 seconds) | Place traffic cone on the mat (opposite side from leash). Close door and step back away from the front of the pen.  Let dog approach, investigate, explore. Record reaction.  After 30 seconds remove the cone from the pen. | Interaction with confident/neutral posture OR ignores but stands near it with confident/neutral posture (no signs of avoidance) | 2 |
|  |  | Approach/avoidance OR exploration with lowered posture OR ignores and does not come near with confident/neutral posture | 1 |
|  |  | Afraid/avoid/aggression, increase distance AND no approach or very brief interaction with lowered posture | 0 |
| 1. **Problem solving**   (up to 30 seconds) | Show a treat. Making sure the dog is watching, place the treat under an upside-down bowl (e.g. transparent plastic food container). Close door and step back away from the front of the pen.  After 30 seconds remove the bowl from the pen and allow dog to eat treat (if unsolved). | Accomplishes task (moves bowl and eats treat) | 2 |
|  |  | Interested/investigates for a majority of time OR lifts/nudges, but does not accomplish | 1 |
|  |  | Does not interact with bowl or attempt the task OR afraid of bowl | 0 |
| 1. **Squeaky toy**   (and habituation) | 1. Show dog toy, squeak it and record immediate reaction. 2. Hold squeaky toy out in front of you and squeak twice, then move in diamond shaped pattern with two squeaks per corner (left, right, up and down) for a total of 10 squeaks. Re-score reaction on final squeak. | Interacting, confident, engaged and/or playful OR ignoring confident/neutral | 2 |
|  |  | Approach/interact cautiously OR interest from distance (e.g. head tilt) | 1 |
|  |  | Afraid/avoiding or aggressive, increase distance | 0 |
|  |  | Interacting, confident, engaged and/or playful OR ignoring confident/neutral | 2 |
|  |  | Approach/interact cautiously OR interest from distance (e.g. head tilt) | 1 |
|  |  | Afraid/avoiding or aggressive, increase distance | 0 |
| 1. **Ball toy**   (up to 30 seconds) | Show a ball toy, drop from approximately 10cm above the mat, then stop with hand to keep in place on the mat. Close door and step back away from the front of the pen.  After 30 seconds remove ball from pen. | Interacting, engaged explores confident for majority of time or plays with the ball | 2 |
|  |  | Approach cautiously OR brief exploration/not interested/ignores with confident/neutral posture | 1 |
|  |  | Afraid/avoiding or aggressive, increase distance | 0 |
| 1. **Artificial dog**   (30 seconds) | Place artificial dog (realistic-like Boston terrier figurine 40 cm height) on the mat at the front of the run. Close door and step back away from the front of the pen.  Record initial reaction.  After 30 seconds remove the fake dog from the pen. | Interaction with confident/neutral posture OR ignores but stands near it with confident/neutral posture (no signs of avoidance) | 2 |
|  |  | Approach/avoidance OR exploration with lowered posture OR ignores and does not come near with confident/neutral posture | 1 |
|  |  | Afraid/avoid, increase distance AND no approach or very brief interaction with lowered posture OR shows aggression | 0 |
|  | Does the dog show any aggression towards the fake dog? | No | 1 |
|  |  | Yes | 0 |
| 1. **Umbrella** | 1. With pen door open, stand in the opening making no eye contact, and slowly open and close a small umbrella and record dog’s behavior. 2. Repeat for habituation, open max 10 times (cease if dog appears to be stressed). Re-score reaction   Does dog show any aggression towards the umbrella? | Interacting, confident, engaged and/or playful OR not interested/ignores, but in close proximity with confident/neutral posture | 2 |
|  |  | Approach cautiously OR interest from distance (e.g., head tilt) | 1 |
|  |  | Afraid, deflecting, increase distance | 0 |
|  |  |  |  |
|  |  | Interacting, confident, engaged and/or playful OR not interested/ignores, but in close proximity with confident/neutral posture | 2 |
|  |  | Approach cautiously OR interest from distance (e.g., head tilt) | 1 |
|  |  | Afraid, deflecting, increase/maintain distance | 0 |
|  |  | No | 1 |
|  |  | Yes | 0 |
| 1. **Commands**   (1 minute – up to 30 sec per command) | 1. *Come*: stand at front of the run and calmly encourage the dog to come with treat in hand. 2. Offer dog a treat 3. *Sit*: crouch, or lower to eye level, in front of dog showing the dog a treat and luring to sit 4. Offer dog a treat | Dog goes to tester, relaxed friendly | 2 |
|  |  | Dog goes to tester, cautious, signs of appeasement, lowered posture | 1 |
|  |  | Dog ignores or afraid, does not go to tester | 0 |
|  |  | Dog eats the treat | 1 |
|  |  | Dog does not eat the treat | 0 |
|  |  | Dog interested, follows the treat with nose and/or sits | 2 |
|  |  | Dog interested but not really following, engaging with person, cautious | 1 |
|  |  | Dog ignores or afraid/aggressive, does not come within reach | 0 |
|  |  | Dog eats the treat | 1 |
|  |  | Dog does not eat the treat | 0 |
| 1. **Loop** l**eash** (habituation) | Calmly pick up slip leash and invite dog to investigate it.  Open large loop and gently attempt to go over dog’s head, while offering treats through the loop.  Keep loop large at all times, never close it around dog’s neck.  Slip it off and allow dog to eat the treat. Record dog’s reaction.  Does the dog show any aggression towards the tester? | Approach, interested, confident/neutral posture when slipping on leash | 2 |
|  |  | Cautious, reluctant, freeze/deflect when slipping on leash | 1 |
|  |  | Afraid, increase distance, aggressive or does not come within reach | 0 |
|  |  | Eats treat | 1 |
|  |  | Does not eat treat | 0 |
|  |  | No | 1 |
|  |  | Yes | 0 |
| **Test is complete. Remove all objects from the pen and close the door.** | | | |

**Section 5: Test-retest - analysis of residuals for LMM**

Test-retest reliability: dependent variable AT_tot (approach test score total) analysis of residuals including (A) normality of residuals, (B) QQ-plot and (C) scatterplot of residuals fitted to the model against the predicted values.


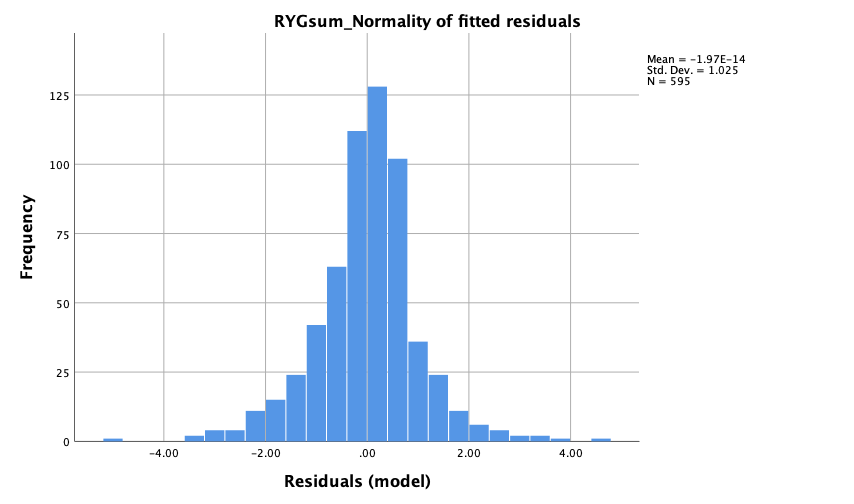
(A)


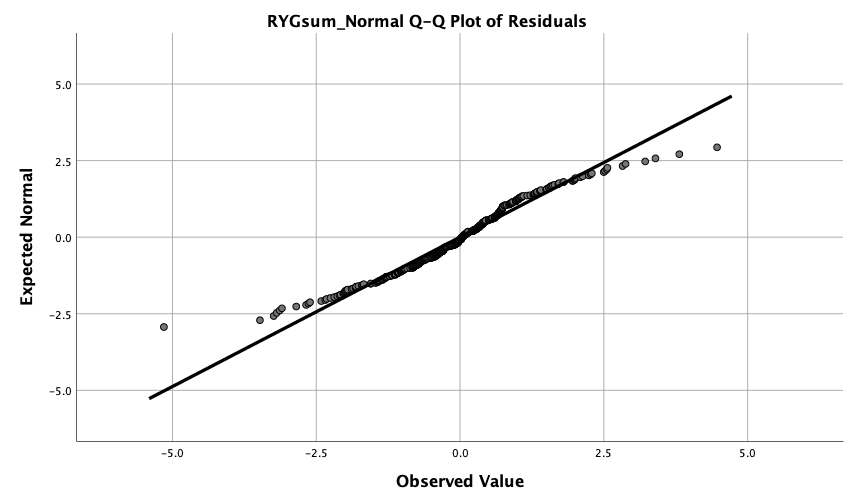
(B)


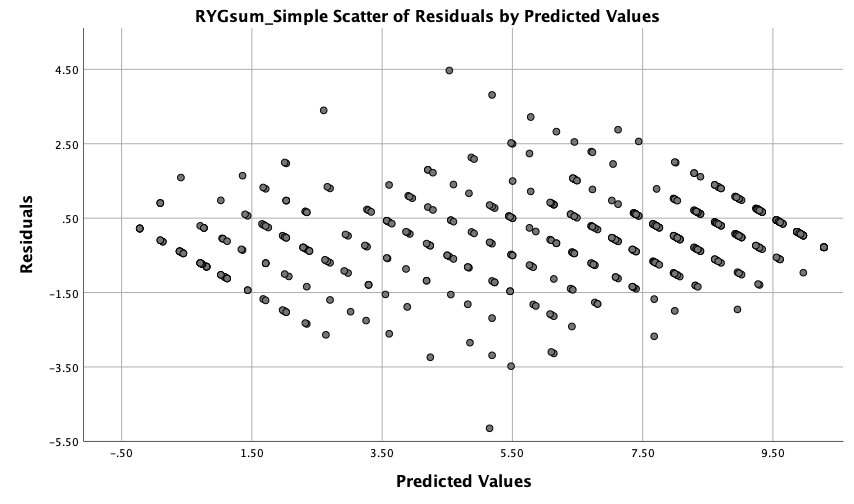
(C)

**Section 6: EFA additional results**

1. Analysis of residuals for each of the four factor scores extracted by the EFA to check model assumptions are met, including (A) normality of residuals, (B) QQ-plot and (C) scatterplot of residuals fitted to the model against the predicted values.

Factor 1_Food motivation


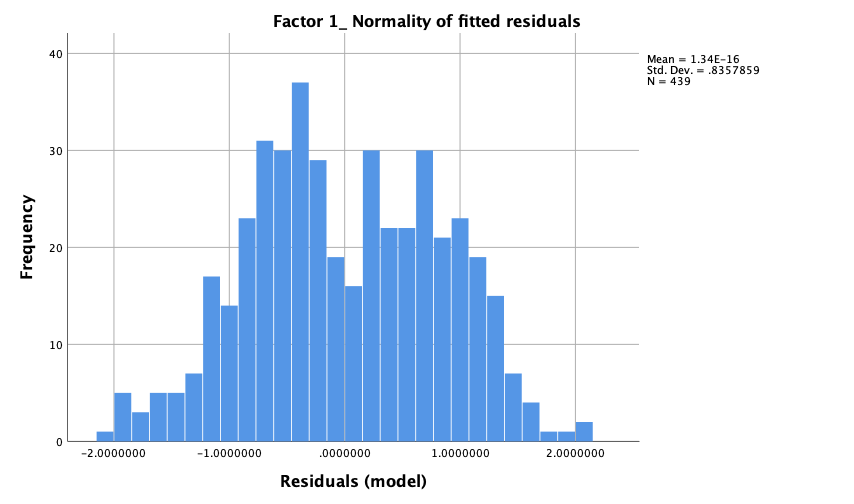


(A)


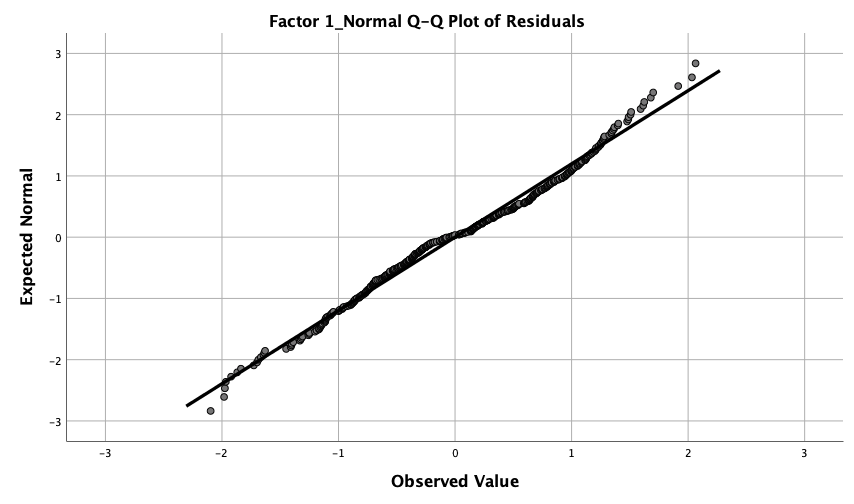
(B)


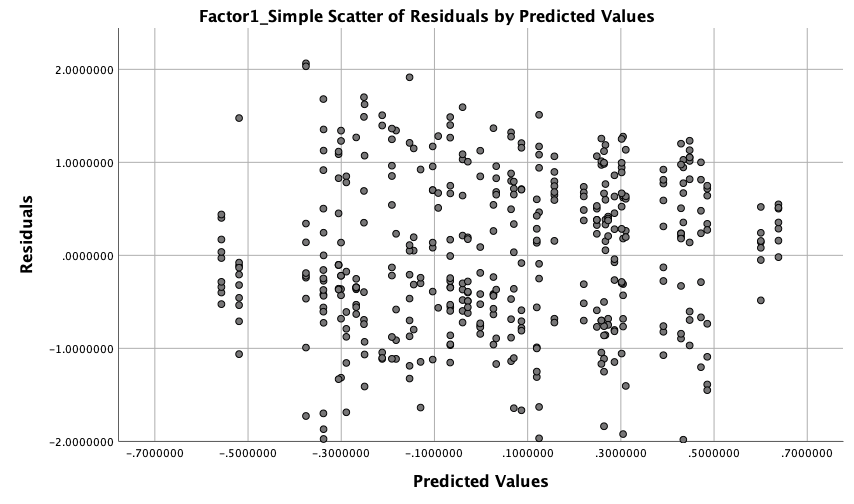
(C)

Factor 2_Sociability


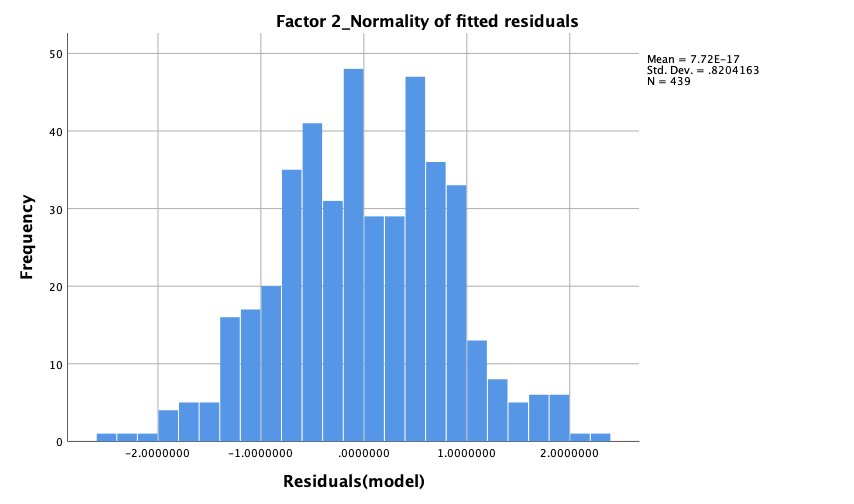
(A)


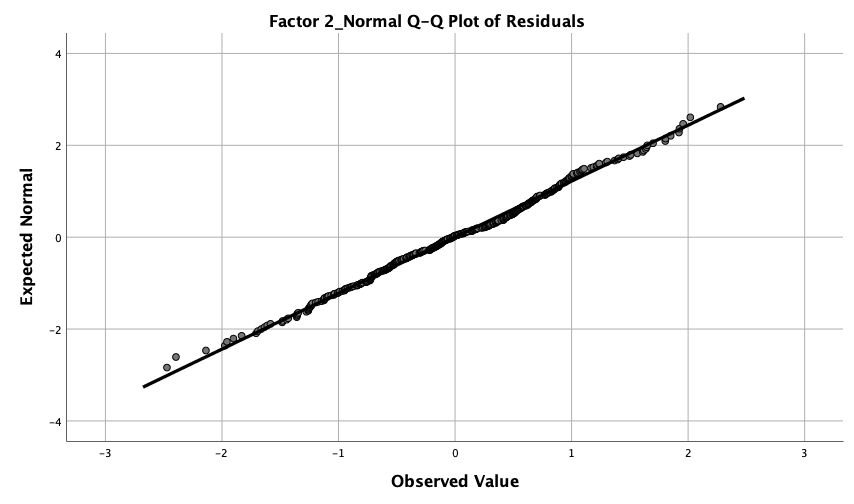
(B)


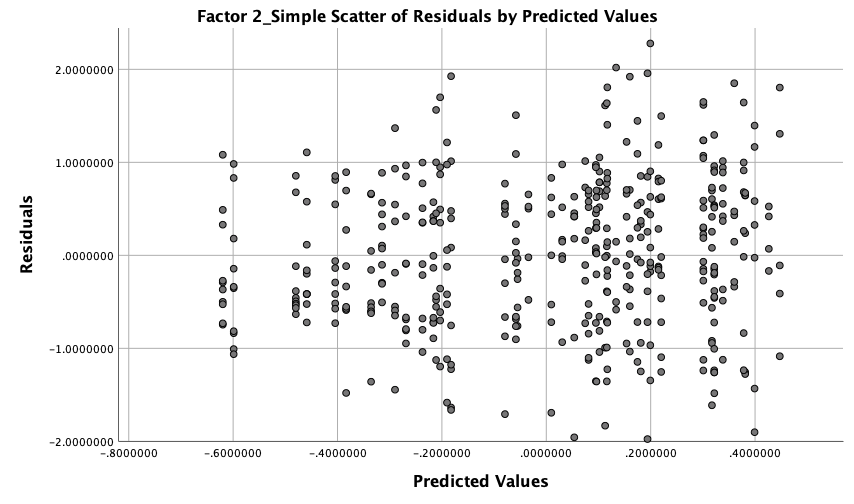
(C)

Factor 3_Boldness


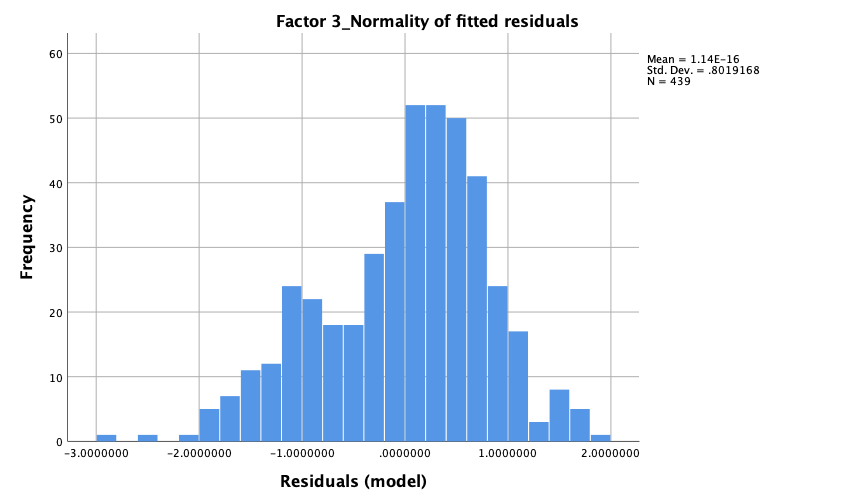
 (A)


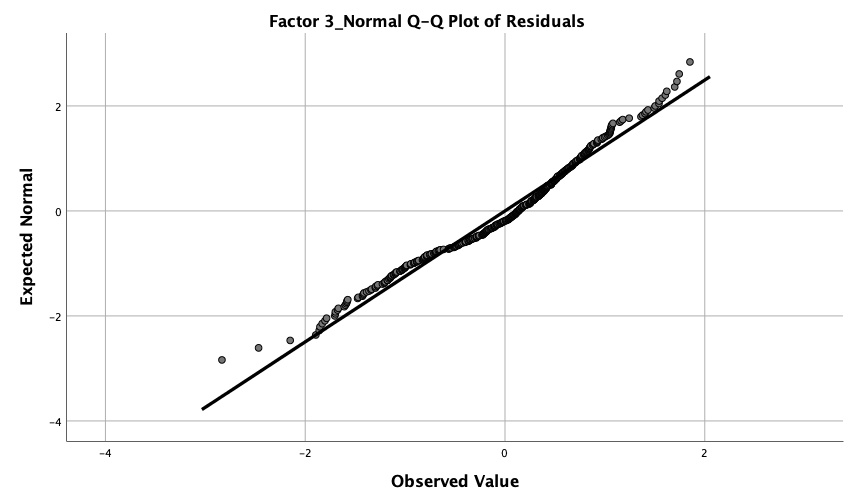
(B)


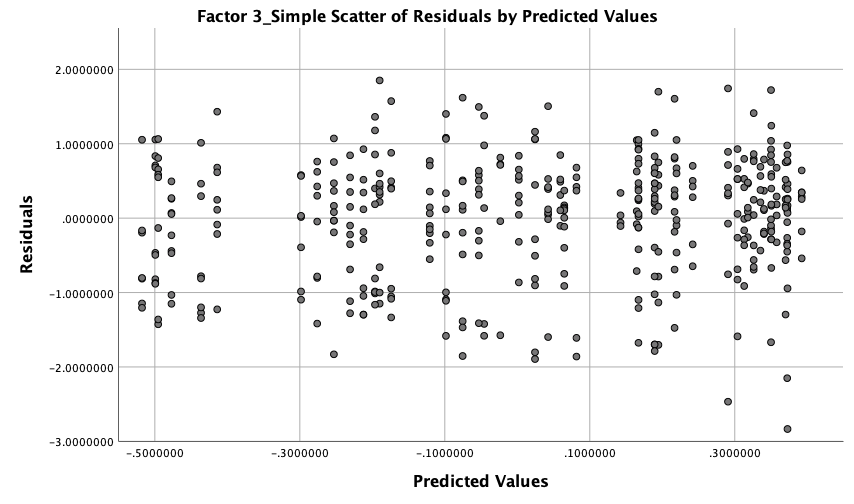
(C)

Factor 4_Responsiveness


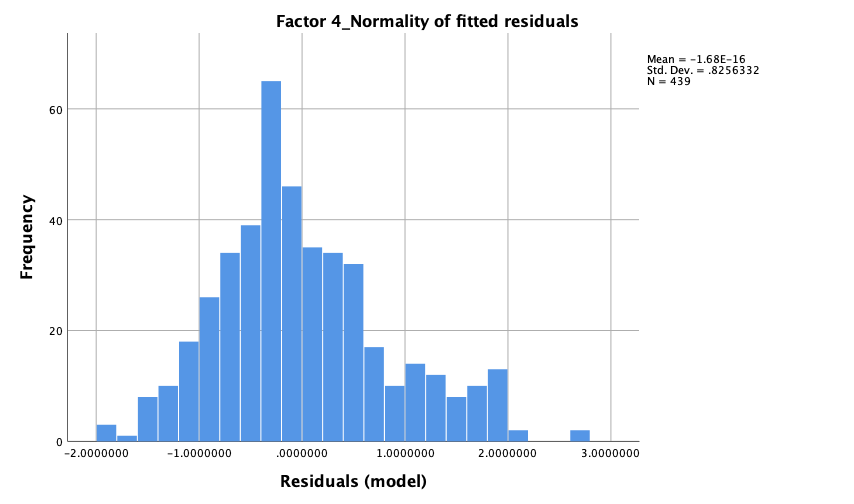
(A)


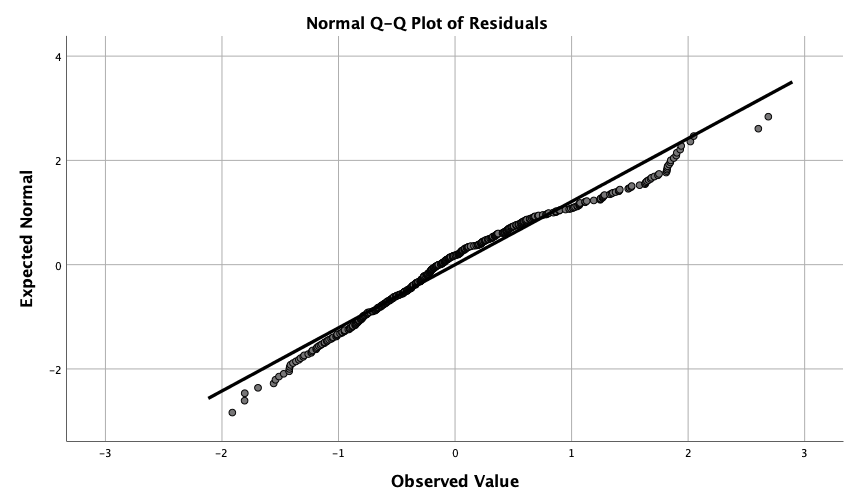
(B)


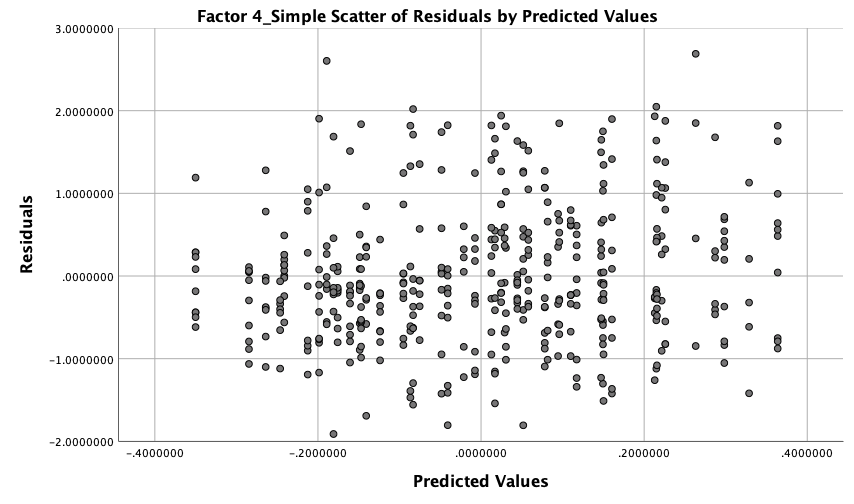
(C)

1. Scree plot for EFA factor extraction (eigenvalue >1)


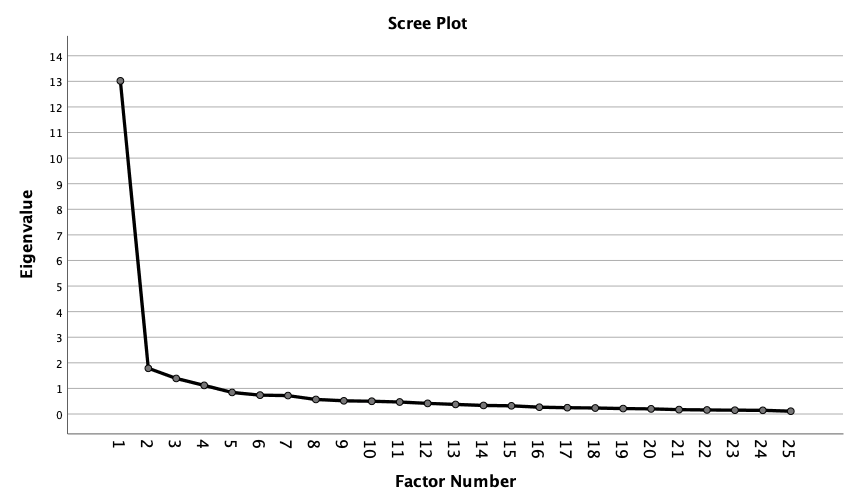


1. Estimates of fixed effects tables for each LMM with the four main component scores extracted from the EFA as dependent variables and fixed effects: tester and reactivity test day (RT_day). Facility as random effect. Empty lines are reference categories

Factor 1_Food motivation

| Parameter | Estimate | Std. Error | df | t | Sig. | 95% Confidence Interval | |
| --- | --- | --- | --- | --- | --- | --- | --- |
|  |  |  |  |  |  | Lower Bound | Upper Bound |
| Intercept | .045297 | .139373 | 27.820 | .325 | .748 | -.240280 | .330874 |
| [Tester=1] | -.031052 | .177111 | 24.068 | -.175 | .862 | -.396537 | .334432 |
| [Tester=2] | . | . | . | . | . | . | . |
| [RT_day=1] | -.037840 | .081780 | 412.834 | -.463 | .644 | -.198598 | .122918 |
| [RT_day=2] | . | . | . | . | . | . | . |

Factor 2_Sociability

| Parameter | Estimate | Std. Error | df | t | Sig. | 95% Confidence Interval | |
| --- | --- | --- | --- | --- | --- | --- | --- |
|  |  |  |  |  |  | Lower Bound | Upper Bound |
| Intercept | -.031689 | .124555 | 28.784 | -.254 | .801 | -.286516 | .223138 |
| [Tester=1] | .076471 | .157059 | 24.110 | .487 | .631 | -.247604 | .400546 |
| [Tester=2] | . | . | . | . | . | . | . |
| [RT_day=1] | .020905 | .080160 | 413.049 | .261 | .794 | -.136668 | .178477 |
| [RT_day=2] | . | . | . | . | . | . | . |

Factor 3_Boldness

| Parameter | Estimate | Std. Error | df | t | Sig. | 95% Confidence Interval | |
| --- | --- | --- | --- | --- | --- | --- | --- |
|  |  |  |  |  |  | Lower Bound | Upper Bound |
| Intercept | .095637 | .125535 | 27.555 | .762 | .453 | -.161697 | .352971 |
| [Tester=1] | -.163528 | .158724 | 23.335 | -1.030 | .313 | -.491612 | .164556 |
| [Tester=2] | . | . | . | . | . | . | . |
| [RT_day=1] | .022304 | .078392 | 412.223 | .285 | .776 | -.131794 | .176402 |
| [RT_day=2] | . | . | . | . | . | . | . |

Factor 4_Responsiveness

| Parameter | Estimate | Std. Error | df | t | Sig. | 95% Confidence Interval | |
| --- | --- | --- | --- | --- | --- | --- | --- |
|  |  |  |  |  |  | Lower Bound | Upper Bound |
| Intercept | -.098079 | .098609 | 30.956 | -.995 | .328 | -.299205 | .103047 |
| [Tester=1] | .136867 | .120763 | 22.895 | 1.133 | .269 | -.113015 | .386748 |
| [Tester=2] | . | . | . | . | . | . | . |
| [RT_day=1] | .065459 | .080233 | 412.547 | .816 | .415 | -.092258 | .223176 |
| [RT_day=2] | . | . | . | . | . | . | . |
